# Potential efficacy of mitochondrial genes for animal DNA barcoding: a case study using eutherian mammals

**DOI:** 10.1186/1471-2164-12-84

**Published:** 2011-01-28

**Authors:** Arong Luo, Aibing Zhang, Simon YW Ho, Weijun Xu, Yanzhou Zhang, Weifeng Shi, Stephen L Cameron, Chaodong Zhu

**Affiliations:** 1 Key Laboratory of Zoological Systematics and Evolution, Institute of Zoology, Chinese Academy of Sciences, Beijing 100101, PR China; 2 Graduate University of Chinese Academy of Sciences, Beijing 100049, PR China; 3 College of Life Sciences, Capital Normal University, Beijing 100048, PR China; 4 School of Biological Sciences, University of Sydney, Sydney NSW 2006, Australia; 5 Zhongbei College, Nanjing Normal University, Nanjing 210046, PR China; 6 UCD Conway Institute of Biomolecular and Biomedical Sciences, University College Dublin, Dublin 4, Ireland; 7 Australian National Insect Collection, CSIRO Entomology, Canberra ACT 2601, Australia

## Abstract

**Background:**

A well-informed choice of genetic locus is central to the efficacy of DNA barcoding. Current DNA barcoding in animals involves the use of the 5' half of the mitochondrial cytochrome oxidase 1 gene (*CO1*) to diagnose and delimit species. However, there is no compelling *a priori *reason for the exclusive focus on this region, and it has been shown that it performs poorly for certain animal groups. To explore alternative mitochondrial barcoding regions, we compared the efficacy of the universal *CO1 *barcoding region with the other mitochondrial protein-coding genes in eutherian mammals. Four criteria were used for this comparison: the number of recovered species, sequence variability within and between species, resolution to taxonomic levels above that of species, and the degree of mutational saturation.

**Results:**

Based on 1,179 mitochondrial genomes of eutherians, we found that the universal *CO1 *barcoding region is a good representative of mitochondrial genes as a whole because the high species-recovery rate (> 90%) was similar to that of other mitochondrial genes, and there were no significant differences in intra- or interspecific variability among genes. However, an overlap between intra- and interspecific variability was still problematic for all mitochondrial genes. Our results also demonstrated that any choice of mitochondrial gene for DNA barcoding failed to offer significant resolution at higher taxonomic levels.

**Conclusions:**

We suggest that the *CO1 *barcoding region, the universal DNA barcode, is preferred among the mitochondrial protein-coding genes as a molecular diagnostic at least for eutherian species identification. Nevertheless, DNA barcoding with this marker may still be problematic for certain eutherian taxa and our approach can be used to test potential barcoding loci for such groups.

## Background

DNA barcoding is an identification approach that uses short DNA sequences from a standardized region of the genome as a molecular diagnostic in species identification. Despite being extremely controversial (e.g., [1-5]), an increasing number of projects are attempting the DNA barcoding of diverse eukaryotic species, especially following the launch of the Consortium for the Barcode of Life (CBOL) [6] in 2004. An ideal DNA barcode should allow fast, reliable, automatable, and cost-effective species identification by users with little or no taxonomic experience [7-9]. Identifications are usually made by comparing unknown sequences against known species DNA barcodes via distance-based tree construction [7,10,11], alignment searching (e.g., BLAST; [12,13]), or methods recently proposed such as the characteristic attribute organization system (CAOS) [14], decision theory [15], and the back-propagation neural network (BP-based species identification) [16].

One of the issues central to the efficacy of DNA barcoding is the selection of a suitable barcode [17]. Interspecific variability in this region should be clearly greater than intraspecific variability, the so-called "barcoding gap"; a threshold value for the magnitude of interspecific variation being 10 times that of intraspecific variation has been proposed as being diagnostic of species-level differences [7,11,17]. Additionally, given that DNA barcoding aims to identify species efficiently, the use of a single barcode marker is preferable (cf. the multi-barcode approach applied in plants [18,19]).

A barcode from the mitochondrial (mt) genome should represent the most effective single-locus marker because of it smaller population size relative to the nuclear genome, which increases the overall concordance between the gene tree and the underlying species tree [20,21]. Accordingly, there has been considerable attention on the use of the mt genome as the source of a barcode locus in animals. The mt genomes of almost all bilaterian animals contain 13 protein-coding genes (PCGs) which encode proteins involved in the oxidative phosphorylation machinery: cytochrome oxidase subunits 1, 2, and 3 (*CO1 *to *CO3*); cytochrome b subunit (*CytB*), NADH dehydrogenase subunits 1, 2, 3, 4, 4L, 5, and 6 (*ND1 *to *ND6*, *ND4L*), and ATPase subunits 6 and 8 (*ATP6 *and *ATP8*). The mt genome also contains 2 ribosomal RNA genes (*16S *and *12S*) and 22 transfer RNA genes. One confounding issue with the use of mt genes in any form of molecular systematics or diagnostics is the widespread nuclear integration of mtDNA resulting in nuclear mitochondrial pseudogenes, or NUMTs, which could introduce serious ambiguity into DNA barcoding [22,23]. However, mtDNA still offers several advantages compared with nuclear DNA: rapid evolution, limited exposure to recombination, lack of introns, and high copy number. These characteristics of mtDNA are important for routine amplification by polymerase chain reaction (PCR) and use as a molecular marker for lower-level questions [7,17,24].

Till now, the most widely used DNA barcode locus for animal taxa is approximately 650 bp from the 5' end of *CO1 *comprising about 40% of the total gene. Although *CO1 *has long been used in animal molecular systematics, initially there was no compelling *a priori *reason to focus on this specific gene among the 13 mt PCGs for DNA barcoding. Indeed, Hebert et al. [7] gave no comparison of the utility of *CO1 *with other mt genes. In practice, *CO1 *has often been used to study relationships of closely related species or even to study phylogeographic groupings within species because of its high level of diversity (e.g., [25,26]). However, the *CO1 *fragment initially chosen for barcoding does have the advantage of being flanked by two highly conserved "universal" primer sites for PCR [7,27,28], which has been helpful for automating the collection of DNA barcodes from a diverse range of organisms.

There have been cases in which the universal *CO1 *DNA barcode has been highly successful in species identification. For example, an identification rate of 100% was achieved in a study of 260 species of North American birds [11]. In contrast, a relatively low success rate (< 70%) was achieved in identifying 449 species of flies (Diptera), owing to an extensive overlap between intra- and interspecific variability [29]. Variability between benthic cnidarian species was found to be very low, with 94.1% of species pairs showing a < 2% difference in their DNA sequences [30]. *CO1 *exhibits significant rate variation within plethodontid salamanders, indicating that genetic distance does not provide a good indication of the time since speciation in this group [31]. Finally, Roe and Sperling [28] found that there was no single optimally informative 600 bp region across the *CO1-CO2 *region, and the universal DNA barcoding region was no better than other regions across these two genes.

Therefore, it is still necessary to search for alternative DNA barcodes to avoid an exclusive reliance on *CO1*. Given the increasing availability of complete mt genomes from a range of taxa, marker choice is no longer constrained by the accessibility of universal primers [31]. Among the mt genes, the 13 PCGs are potentially better targets for DNA barcoding owing to lower levels of insertions and deletions (indels), which can complicate the process of sequence alignment [7], than are found in alignments of ribosomal RNA genes which have also been proposed as species-level markers [32,33]. Recently, there have been certain studies that evaluated no more than 4 already proposed regions as DNA barcodes for amphibians, primates, birds, and other groups [33-37], but the majority of the mt PCGs have never been evaluated for their barcoding utility. Further, the evaluation of alternative barcode regions has focused on groups where *CO1 *has already been shown to underperform (e.g. [33,34]) rather than test if any other gene may be superior. This stands in contrast to the systematic investigations into phylogenetic performance (e.g., [31,38]) or adaptive evolution [39] of most mt PCGs, and the approach of the fungal barcoding protocol [40].

We here present a bioinformatics approach to evaluate the efficacy of each of the 12 mt PCGs (*ND6 *was excluded because of its situation on the opposing light strand and the presence of many indels) along with the universal *CO1 *barcoding region as potential DNA barcodes for eutherian mammals. For this major animal group, there are a large number of mt genomes publicly available, including multiple samples from many species, and a well-defined taxonomic system. Our evaluation of each gene profile includes the following: (1) the number of barcode species recovered in the neighbour-joining (NJ) tree, (2) sequence variability within and between species, (3) resolution to higher taxonomic levels, and (4) best-fit evolutionary model and DNA saturation.

## Results

### Similar numbers of recovered barcode species

Table 1 shows the summed numbers of species recovered as monophyletic groups (henceforth referred to as "barcode species") in NJ trees under the Kimura-Two-Parameter (K2P) model [41]. Although numbers of potential barcode species varied among the 14 NJ trees (ranging from 52 for the whole genome to 62 for whole *CO1 *and the *CO1 *barcoding region), species that were not recovered represented only a small proportion of the total species, with recovery rates ranging from 91.53% for *ND1 *to 96.15% for the genome. In each NJ tree, there were almost always the same four species that could not be recovered (*Bos indicus*, *Bos taurus*, *Chlorocebus pygerythrus*, and *Ursus arctos*). *Bos indicus *was recovered as a barcode species only in two trees derived from profiles of the *CO1 *barcoding region and *ND4L *respectively, and had only one representative sequence in profiles of the *ATP6*, *ATP8*, and the whole genome. *Bos taurus*, having more than 130 representative sequences in the 14 profiles, was always clustered with *Bos indicus *and *Bos javanicus *(NC_012706) in the 14 NJ trees, which rendered it non-monophyletic; only a subset of *Bos taurus *sequences formed a monophyletic group. The two sequences of *Chlorocebus pygerythrus *did not form a monophyletic group in any of the NJ trees. The two sequences of *Ursus arctos *almost always formed a paraphyletic group with respect to the monophyletic cluster of *Ursus maritimus*, although both of them were recovered as barcode species in trees derived from the *ND4 *and *ATP8 *profiles. Other unrecovered species (*Bubalus bubalis*, *Cervus nippon*, and *Elephas maximus*) were non-monophyletic in trees derived from profiles of the *CO1 *barcoding region, *ND1*, *ATP6*, and *ATP8*. The recovered barcode species ranged from *Acinonyx jubatus *represented by two sequences with no other congeneric species to *Canis lupus *represented by more than 250 sequences, to *Balaenoptera acutorostrata *with 7 congeneric species as well. Tree files are available on request.

**Table 1:** Details of the 14 profiles in this study

Profile	Length (bp)	Seq. No.	Genera				Species			
			
			No.	Seq. No.	Cohesive group No.	Rate (%)	No.	Seq. No.	Barcode Sp. No.	Recovery rate (%)
*ATP6*	705	917	35	616	23	65.71	59	723	55	93.22
*ATP8*	216	921	35	621	24	68.57	60	729	56	93.33
*CO1*	1,554	909	35	613	23	65.71	62	718	58	93.55
*CO2*	693	913	35	619	25	71.43	59	721	55	93.22
*CO3*	834	885	35	591	22	62.86	61	695	57	93.44
*CytB*	1,143	911	34	615	21	61.76	60	723	56	93.33
*ND1*	966	917	35	620	23	65.71	59	724	54	91.53
*ND2*	1,062	912	35	616	22	62.86	59	719	55	93.22
*ND3*	363	912	35	615	23	65.71	58	719	54	93.10
*ND4*	1,431	903	35	609	23	65.71	58	710	55	94.83
*ND4L*	294	913	35	617	22	62.86	58	719	55	94.83
*ND5*	1,866	910	35	615	22	62.86	59	717	55	93.22
Barcoding region	648	909	35	613	23	65.71	62	718	58	93.55
genome	11,127	847	34	565	22	64.71	52	655	50	96.15

For each profile, sequence length and the number of sequences (except the two outgroups) are shown. 'Genera' denotes the number of instances with sequences representing at least two different species in each genus. 'Species' denotes the number of instances having at least two sequences representing each species

### Homogeneous intra- and interspecific variability

**Figure 1:**
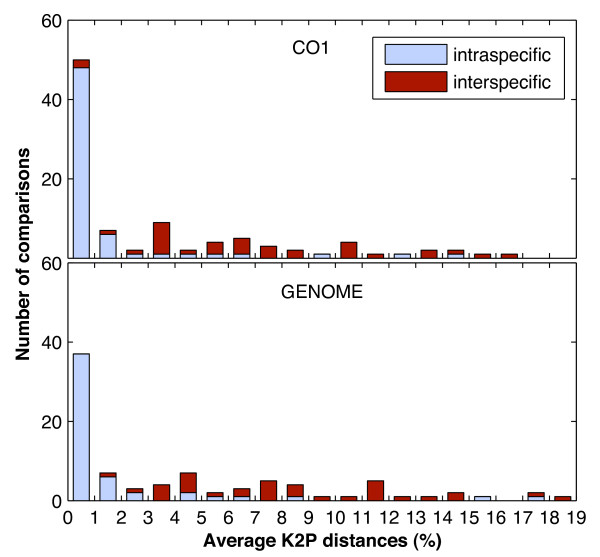
**Average distances versus intra- and interspecific comparisions from the *CO1 *and genome profiles**. The x-axis represents K2P distance values (%) and the y-axis represents the number of comparisons. The number of comparisons indicates either the number of species compared (intraspecific comparisons, blue) or the number of genera compared (interspecific comparisons, red).

**Figure 2:**
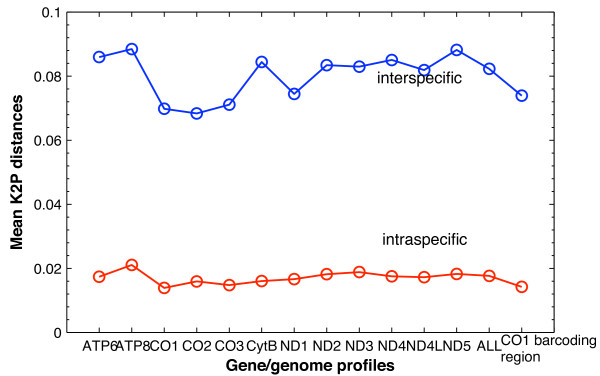
**Mean intra- and interspecific distances from the 14 gene/genome profiles**. Markers in red denote mean intraspecific distances, while those in blue denote mean interspecific distances. The 13 gene regions are listed on the x-axis, with the genome represented by "ALL".

Average K2P distances for each species or genus were here used to determine whether there were differences in sequence variability among different genes. For most species in the 13 gene profiles, average intraspecific distances were less than 3% (with certain exceptions including *Ammotragus lervia*, *Chlorocebus pygerythrus*, and *Galeopterus variegatus*; Figure 1; Additional file 1), resulting in similar mean distances for each of the 13 genes (~1.5%; Figure 2). Average intraspecific distances for *Ursus arctos *were often greater than 2%. An ANOVA-Tukey test showed that there was no significant difference in the average intraspecific distances among the 13 different genes (P = 0.998) or between any gene pair. Average interspecific distances were more than 3% within most genera, with some exceptions including the two genera of *Aotus *and *Eubalaena *for which the K2P distances were always less than 2.6% or even zero across the 13 gene profiles (Figure 1; Additional file 1). Although the mean interspecific distances for the 13 genes differed slightly from each other (ranging from ~7% to ~9%; Figure 2), there was no significant difference in the average interspecific distances among the 13 different genes (P = 0.598) or between any gene pair. As average intraspecific distances for *Bos indicus *and *Bos taurus *were always less than 0.3%, interspecific distances for any species pair of *Bos indicus*, *Bos taurus*, and *Bos javanicus *were generally less than 2% in these gene profiles, consistent with their non-monophyly in the NJ species-recovery tests.

For the average K2P distances from the genome profile, mean intra- and interspecific distances were ~1.76% and ~8.23%, respectively (Figure 2). Distance biases found for different species or genera were similar to those in separate gene profiles (Figure 1; Additional file 1). There were no significant differences between the inter- or intraspecific distances from the genome profile and those from any of the 13 gene profiles (all P > 0.99).

**Figure 3:**
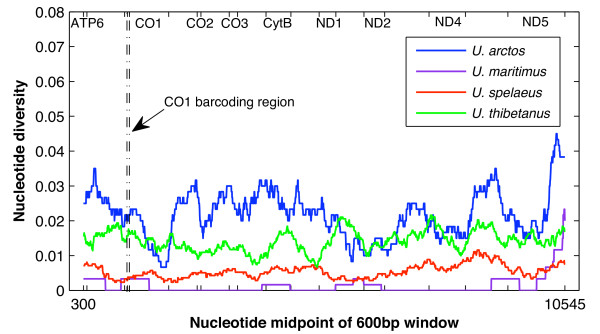
**Nucleotide diversity of four species of the genus *Ursus***. The x-axis represents nucleotide midpoints of the 600 bp window. The range of each gene is annotated at the top of the plot with a pair of ticks.

With the genus *Ursus *as an example, Figure 3 and Additional file 2 show the uncorrected intra- and interspecific distances from sliding-window analyses based on concatenated genome sequences (see Methods for details). There are no values for *ATP8*, *ND3*, and *ND4L *because the lengths of these genes were less than the window size (600 bp). Variability of intra- and interspecific distances across different gene regions was relatively small, e.g. the extinct species *U. spelaeus *(red line in Figure 3), whereas variability among different species or species pairs was more obvious. Taking the *CO1 *barcoding region as an example, nucleotide diversities for the four species ranged from ~0.002 (*U. maritimus*, purple) to ~0.02 (*U. arctos*, blue) (Figure 3). For the nucleotide diversity of *U. arctos *(blue line), clear variability exists among different gene regions. However, if the universal *CO1 *barcoding region was taken as the benchmark, there are other regions (*CO1*, *CO2*, *CO3*, *CytB*, *ND1*, *ND5*) at which *U. arctos *has similar patterns of evolutionary distances.

### Poor resolution at higher taxonomic levels

The summed numbers of cohesive groups at the genus level are shown in Table 1. There were more than 20 but fewer than 26 cohesive groups in the 14 NJ trees, which represented moderate proportions of the potentially cohesive ones (ranging from 61.76% from the *CytB *profile to 71.43% from the *CO2 *profile).

**Figure 4:**
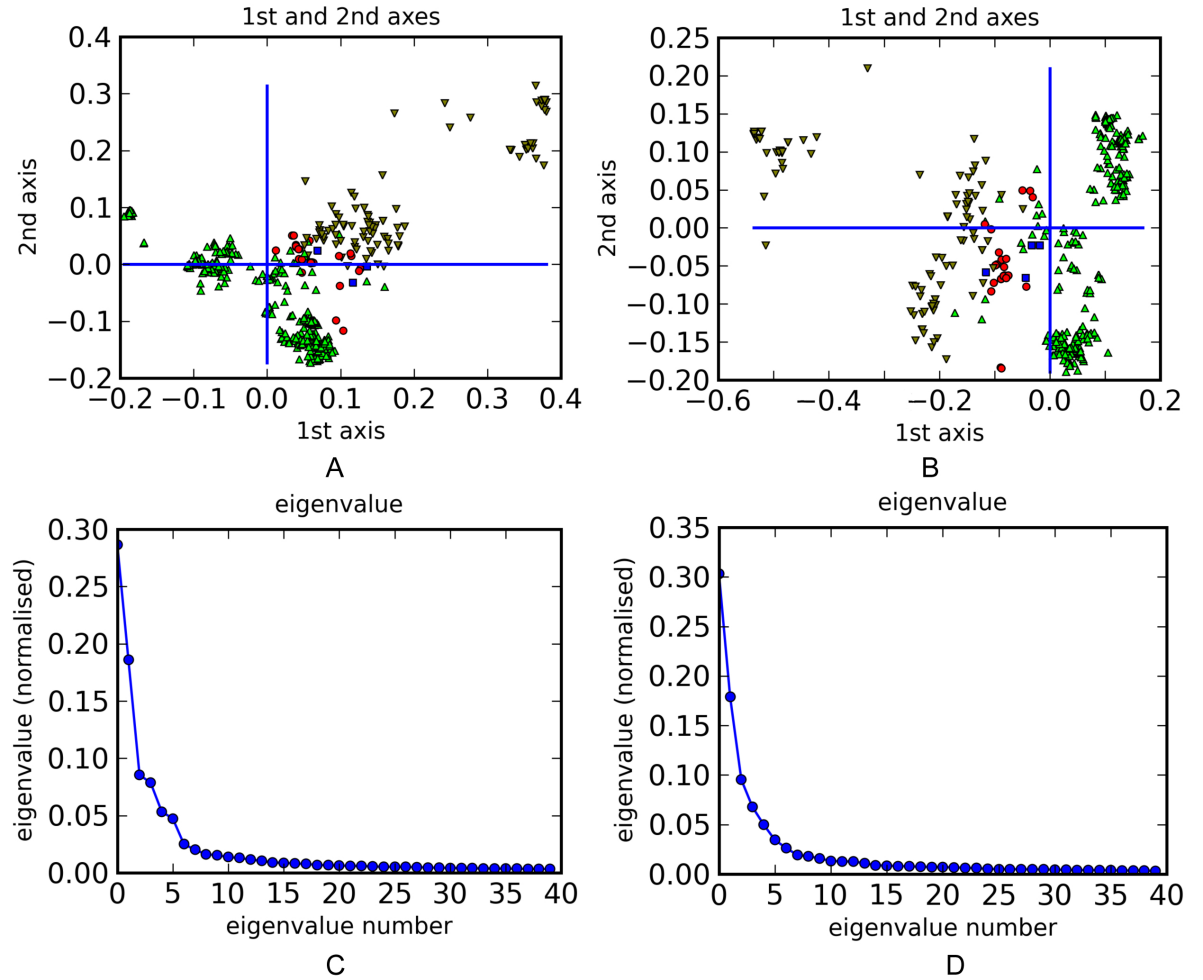
**Grouping results from PCOORD for the *ND2 *profile**. Figure 3A shows the grouping based on nucleotide sequences, and Figure 3B based on amino acid sequences. Symbols indicate: Afrotheria (red circle); Euarchontoglires, (grey down-pointing triangle); Laurasiatheria (green up-pointing triangle); and Xenarthra (blue square). Figure 3C and Figure 3D show the associated eigenvalues from nucleotide and amino acid sequences, respectively.

With principal-coordinates (PCOORD) analysis (see Methods for details), Figure 4 shows the grouping at the super-ordinal level of the first two significant dimensions for 912 nucleotide and inferred amino acid sequences of the *ND2 *profile. Although most sequences of the super-order Laurasiatheria (green, up-pointing triangle marker) tended to separate from those of the other three super-orders (Figure 4A, nucleotide sequences; Figure 4B, amino acid sequences), some clustered with sequences belonging to Euarchontoglires (grey, down-pointing triangle marker), Xenarthra (blue, square marker), and Afrotheria (red, circle marker). None of the super-orders represented by taxon sequences fell into a completely distinct cluster. With reference to the eigenvalues, clustering from the first two dimensions (~28% and ~18% respectively in Figure 4C; ~31% and ~18% respectively in Figure 4D) accounted for less than 50% of the total distance information. Resolution of the four super-orders with sequences from the other 12 gene profiles was similar to or worse than that from the *ND2 *profile which showed the highest resolution (see Additional file 3).

### Best-fit model and saturation for distant species

**Figure 5:**
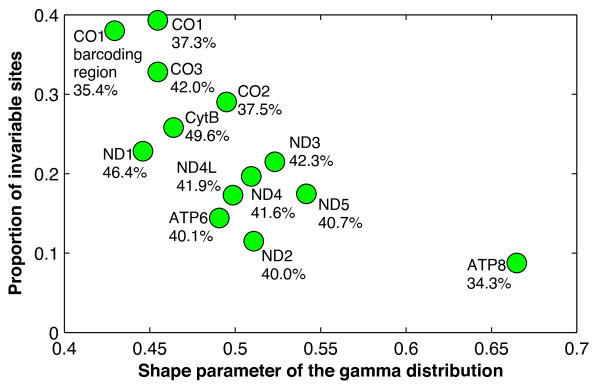
**Estimated values of substitution model parameters for the 13 gene profiles**. The x-axis represents values of shape parameter of the gamma distribution, and the y-axis represents ones of the proportion of invariable sites. GC% values are also shown for each profile.

For the 13 gene profiles, the Bayesian information criterion (BIC) as implemented in ModelTest v.3.7 [42,43] selected the general time-reversible (GTR) model with a proportion of invariable sites (I) and heterogeneity of substitution rates among sites (modelled using a gamma distribution, Г) as their best-fit evolutionary models (Figure 5). The *ATP8 *gene was distinct in having a smaller proportion of invariable sites (0.0877) and less heterogeneity of substitution rates among its 216 sites (shape parameter = 0.6648). The GC% ranged from 34.3% in *ATP8 *to 49.6% in *CytB*.

**Figure 6:**
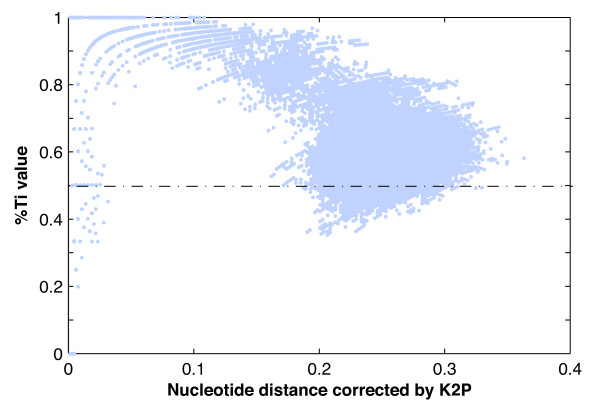
**%Ti values against pairwise K2P distances for the *CO1 *barcoding region profile**. The x-axis represents the K2P distance values, while the y-axis represents %Ti values.

A plot of %Ti values against pairwise K2P distances is shown in Figure 6 for the *CO1 *barcoding region, with those representing pairwise distances of zero excluded. There is no obvious pattern in the %Ti values for near-zero pairwise distances, but as the nucleotide distances increase above a value of 0.1, %Ti values decrease from between 0.8 and 1.0 (for pairwise distances of ~0.1) to between 0.8 and 0.5 (for pairwise distance of ~0.3), indicating saturation for comparisons between genetically distant species. Other gene profiles (Additional file 4) gave similar patterns to those found from the *CO1 *barcoding region, with the exception of *ATP8*. In the plot for *ATP8 *(see Additional file 4A), very low %Ti (~30%) disappeared when pairwise distances were larger than 0.2, whereas values tended to increase when distances exceeded 0.7; having been corrected for multiple hits by the K2P model, distances of some sequence pairs were larger than 1.

## Discussion

Our evaluation of the efficacy of mt genes for animal barcoding has focused on the mt genomes of eutherians. This group affords several advantages as a model group for such an investigation. First, compared with insects and other invertebrates, the taxonomic system of eutherians is relatively clear and complete although certain problems still need to be resolved (e.g., [44]). Second, owing to the large number of complete mt genomes available for this group, we were able to limit our analysis to putatively orthologous genes [23], eliminating the ambiguity caused by any NUMTs that might have entered our dataset had we used all the mt gene sequences available on GenBank. Third, using whole mt genomes we are able to simultaneously evaluate the utility of 12 PCGs as DNA barcodes (versus 4 in the next-largest study [34]), while controlling for variation between individuals because a single genome sequence was used as the source for each of the 12 evaluated genes. Fourth, although mtDNA recombination might occur in certain animals [45,46], it is generally acknowledged that recombination in mtDNA is limited and maternal inheritance is the norm in mammals. Thus, we can reasonably expect that all of the mt genes share the same underlying genealogy, and non-monophyly of species caused by incomplete lineage sorting will affect all mt genes identically. Taking these various issues into account, our investigation is essentially a test of the relative evolutionary information of different mt genes. Finally, while most DNA barcoding studies have focused on extant species within discrete geographical areas [14], the mt genomes in this study have been sequenced from a broad geographical range (Additional file 5). By investigating them we can test DNA barcoding loci on a worldwide scale. Additionally, some of the analyzed mt genomes were from extinct species (e.g., *Ursus spelaeus*; Additional file 5). In spite of the small number of included sequences, we are able to provide a preliminary assessment of the resolution of DNA barcoding for extinct species.

### Universal *CO1 *barcoding region is representative of mitochondrial genes

Results from the first two evaluations (i.e., similar recovery rates based on the NJ tree and homogeneous variability within and between species based on K2P distances) indicate that the 5' end of *CO1*, the standard barcoding region for animals [47], is not only representative of the whole *CO1 *gene but also the 12 mt PCGs, despite the fact that gene lengths ranged from 216 bp of *ATP8 *to 1,866 bp of *ND5*. This finding is consistent with the conclusion of Roe and Sperling [28] that subsections of *CO1*-*CO2 *region (~2.3 kb) have similar performance and that none is significantly better than the others. Min and Hickey [48] showed that the *CO1 *barcoding region provides a quick preview of mt genome composition. Our results from the comparisons between the genome profile and the 13 individual gene regions indicate that the *CO1 *barcoding region is also representative of the efficacy of the mt genome as a whole (the 12 PCGs together in our study). In practice, there is some inconsistency in the specific position and length of the 5' end of *CO1 *used in different barcoding studies [17], which often vary depending on available primers and the ability to amplify specific taxa. With additional results from the sliding-window analyses (Figure 3; Additional file 2), we provide further insight into this by demonstrating that any of the 600 bp fragments from the 5' end of *CO1 *had similar evolutionary patterns. Thus, we suggest that standardization of the exact barcode fragment is necessary only to allow for the automation of barcode collection, not due to the inherent superiority of any given subregion of *CO1*.

The high percentage (93.55%; Table 1) of recovered barcode species and lower mean intraspecific distance (~1.4%) derived from the profile of *CO1 *barcoding region generally suggest that *CO1 *barcoding region is an effective molecular marker in the identification of eutherian species, including extinct species and those distributed across broad geographical scales (e.g., *Mammuthus primigenius *and *Ursus spelaeus*; see Additional file 5). For species not recovered in the NJ tree and those with larger intraspecific distances, possible reasons have been given by Nijman and Aliabadian [37]. Sequenced species of *Bos *might have undergone hybridization or introgression [49,50]; and our results support the mitochondrial paraphyly of brown bears (*Ursus arctos*) with respect to polar bears (*Ursus maritimus*) as demonstrated in previous studies [51,52].

### Overlap between intra- and interspecific variability

For a DNA barcode to be effective, interspecific differences should be clearly, and preferably significantly, greater than intraspecific differences [7,17]. On one hand, with the clear gap between mean intra- and interspeicfic distances (Figure 2), our analyses of variability within and among species generally confirm the potential of mt genes and the *CO1 *barcoding region specifically as suitable DNA barcode loci. On the other hand, intraspecific distances of some species were much larger than the mean intraspecific distance, while interspecific distances of some congeneric species were much smaller than the mean interspecific distance (Figure 1; Additional file 1). Examples include *Ammotragus lervia*, *Chlorocebus pygerythrus*, and *Galeopterus variegatus*, for which average intraspecific distances were very high (greater than 3%). Conversely, interspecific distances for two species of *Aotus *were less than 0.7%, extremely low compared to other barcoding studies. Evolutionary patterns of the three *Bos *species (*B. indicus*, *B. taurus*, and *B. javanicus*) reveal that there is no significant difference between intra- and interspecific distances across the 12 PCGs, which is likely due to hybridization or introgression as discussed above. Therefore, the problem of overlapping variability does exist in some taxa. It is notable that the species for which intraspecific distances were larger than or overlapped with interspecific distances (to congeneric species) tended not to be recovered in NJ trees (e.g., *Bos taurus*, *Chlorocebus pygerythrus*, and *Ursus arctos*). Thus, the problem of overlapping more or less challenges the fundamental basis of DNA barcoding; moreover, our results suggest that none of the other mt genes escapes from this problem. Additionally, we did not find any obvious dependence of the average intraspecific distance on the sample size for each species (Additional file 5; Additional file 6).

While comparing variability within and among species, distances were calculated under the K2P model. When it is used to measure pairwise sequence distances, the K2P model differentiates transitions from transversions [41]. In sliding-window analyses, however, we measured pairwise distance with the average number of nucleotide substitutions per site, as with the uncorrected pairwise distance (*p*-distance) in PAUP* v4.0b10 [53]. This will lead to some discrepancy between the two values; however, this did not influence our comparison among genes in separate analyses.

### Failure to resolve at high taxonomic levels and DNA saturation

One of the major criticisms of DNA barcoding is the species concept that it implements [54]. Considering that most modern species concepts recognise the complex, dynamic relationships between organisms and lineages [54], it seems necessary for DNA barcoding to identify specimens within higher taxonomic groupings while still focusing on species-level identifications. Certain studies have investigated barcoding in this context (e.g., [7,35,55]). Our results show that, compared with the high species-recovery rates of more than 91%, the 14 profiles gave poor resolution at the genus level, with cohesive groups accounting for less than 72% of the potentially cohesive ones. With PCOORD, which can detect grouping of deep branches and has been used in analyses of virus sequence variation [56], we also evaluated resolution to the level of super-order. The four super-orders considered in our analysis represent the major phylogenetic groupings of eutherians. However, in contrast with other studies that achieved good resolution at the levels of phylum and order based on small numbers of sequences [7], species cannot be confidently assigned to the four eutherian super-orders even with the large number of both nucleotide and amino acid sequences included in this analysis (Figure 4; Additional file 3). Thus, our results indicate that DNA barcoding cannot offer good resolution at higher taxonomic levels within eutherians. Accurate species identifications will be dependent upon comprehensive barcode databases, as sequences from unrepresented species cannot be reliably placed into higher taxonomic groups for which sequences from other species are available.

The failure of mtDNA barcoding at higher taxonomic levels is not entirely unexpected, given the likelihood that the eutherian orders diversified over a relatively short timeframe. In each gene profile, given that GTR + I + Г was selected as the best-fit evolutionary model, it is likely that the routinely used K2P model underestimates the number of multiple substitutions at each variable site [57]. In addition, we found that low %Ti values disappeared as the nucleotide distance increased. Both of these signify that phylogenetic information is lost for distantly related species pairs, eventually resulting in a misleading signal. We suggest that the low %Ti for genetic distances near zero (Figure 6; Additional file 4) can be attributed to the fact that, compared with transversions, transitions would be less obvious if the K2P model did not correct for the multiple hits hidden behind transversions for closely related sequence pairs.

## Conclusions

With a large number of mt genomes available for eutherians, our evaluation of the efficacy of DNA barcoding demonstrates that the 5' end of *CO1*, the universal DNA barcode, is a good representative of the mt PCGs. It suggests that any one of the 12 PCGs (other than *ND6*, which was not considered in this study) can be potentially used as a molecular diagnostic for species identification. However, considering the criteria in the CBOL's data standards and guidelines for locus selection [18]: *universality*, *sequence quality and coverage*, and *discrimination*, the universal *CO1 *barcoding region should be the first choice among these mt PCGs. Abundant sequence data from this region are already available for a wide variety of animal species; *CO1 *sequences can also be used in phylogenetic research together with other genes, whereas short genes such as *ATP8 *would be less useful because of the greater impact of saturation. Therefore, our conclusion is generally consistent with that of previous studies in which alternative regions (e.g., *CO1*, *CytB*, *16S*) were compared [34,35,37]. Nevertheless, our results confirm that DNA barcoding still faces the problem of overlap between intra- and interspecific variability for a portion of species in any group. Our analyses also indicate that mtDNA barcoding cannot offer good resolution at higher taxonomic levels, and thus the accuracy of species identifications is linked to the completeness of the DNA barcode database against which unknown sequences are compared.

We have considered 12 mt PCGs together with the universal *CO1 *barcoding region as potential candidates for DNA barcodes in this study. It is true that each gene functions as a biological entity with different evolutionary pressures, yet a single short segment such as the 5' end of *CO1 *may be sufficient for species identification in eutherians. Thus, in the future, although we have analyzed sequence variability of these genes with a 600 bp sliding window, explicit studies of short segments of mt genes need to be done. Of course, DNA barcoding does not need to be limited to mt genes. With the growing availability of sequences from nuclear genes, it is quite likely that some nuclear markers could be effective DNA barcodes, which should be tested for efficacy by methods such as ours in the present study. Furthermore, similar studies can be done to understand the behaviour of potential barcode loci in other large taxonomic groups; at the moment, however, the availability of whole mt genomes from multiple conspecific specimens is limited in most animal groups.

## Methods

### Recovery of mitochondrial genomes and aligned protein-coding genes

A total of 1,179 complete or partial mt genomes were obtained from the National Center for Biotechnology Information (NCBI) Nucleotide Database with resources from GenBank, RefSeq, and others [58], holding almost all currently available (as of September 16, 2009) genome sequences for eutherians except for modern humans. Owing to the large number of mt genomes for humans available, only two (NC_012920 and NC_011137) from RefSeq [59] were used in this study. Additionally, we downloaded another two for metatherians (*Caenolestes fuliginosus *NC_005828 and *Dactylopsila trivirgata *NC_008134) which were used as outgroups in subsequent tree-based evaluation. Where the same genome was obtained from multiple databases, duplicates were culled such that only a single copy was used in analyses.

We partitioned the genomes into 12 of the 13 PCGs; the *ND6 *gene was excluded because of its situation on the opposing light strand and the presence of many indels. Gene sequences that were obviously shorter than most homologous sequences or that contained large ambiguous regions (continuous strings of N's) were removed from each gene profile before alignment. Sequences were aligned based on the inferred amino-acid sequence using Muscle v3.6 [60] with default parameter settings; stop codons were removed from each alignment. Details of genome and gene sequences are shown in Additional file 5.

Aligned sequences that shared accession numbers (i.e. derived from the same genome sequence) were concatenated in the alphabetic order of gene names, resulting in 847 sequences plus two outgroups which we term the "genome profile" for this study. Additionally, the canonical *CO1 *barcode fragment of 648 bp was obtained from the 5'end of the *CO1 *profile, spanning positions 58 to 705 [47]. This region is termed the "*CO1 *barcoding region" in this study and is analyzed alongside the 12 gene profiles and the genome profile. Sequence lengths and the numbers of sequences of the 14 profiles are shown in Table 1.

### Tree-based efficacy of the mitochondrial gene candidates

Prior to tree construction, sequence names were formatted using accession numbers and organism names (genus and species names), which were generally consistent with the nomenclature and taxonomy in the Nucleotide Database for each mt genome (see Additional file 5 for taxonomic information). For all 14 profiles, PAUP* v4.0b10 [53] was used to perform phylogenetic reconstruction using NJ method with the K2P model [41], which model has routinely been used in barcoding studies and was recommended by Barrett and Hebert [55]. We define a species as a recovered "barcode species" if conspecific sequences, defined by their taxonomic assignment in the Nucleotide Database, formed a monophyletic cluster. The number of recovered barcode species was summed for each NJ tree derived from each profile to give the proportion of total species that was recovered by each mt gene or genome.

### Evolutionary patterns of mitochondrial genes

To study the evolutionary patterns of nucleotide divergence, we compared variability within and between species across the 14 profiles. First, pairwise distances under the K2P model were calculated by PAUP* v4.0b10 [53] for conspecific sequences from ~60 species in each profile (Table 1). As some of these species had more than two representative sequences, the average of the pairwise distances was estimated for each species. Second, for any species that had sequences from multiple conspecific representatives as well as from congeneric species in the profile, we constructed a consensus sequence for each species using Mesquite v2.6 [61]. Conspecific sequence variability was summarized by IUPAC codes. Third, after pairwise interspecific K2P distances for each set of congeneric sequences, among which some were consensus sequences as described above, were computed with PAUP* v4.0b10 [53], the average interspecific distances were estimated for each of ~35 genera in 14 profiles (Table 1). The "missdist = infer" option in PAUP* v4.0b10 [53] was used to infer distances at ambiguous sites by distributing them proportionally to unambiguous changes. Statistical significance of differences in the intra- or interspecific average distances among different profiles respectively was estimated by one-way ANOVA followed by a Tukey test using PASW v18 [62].

To visualize genetic variability within and between species, we performed sliding-window analyses with DnaSP v5.10 [63,64]. We used the genus *Ursus *as a special case due to the intensive sequencing efforts on this genus, with four of its five species represented by at least two mt genome sequences for each species. Sloth bear (*Melursus ursinus*) and sun bear (*Helarctos malayanus*) are excluded, although some studies have classified them into the genus *Ursus *[65]. For the sake of convenience, we analysed all 19 of the concatenated sequences available from the genome profile (Table 2). Nucleotide diversity (i.e., average pairwise number of nucleotide substitutions per site) (equation 10.5 of [66]; [64,67]) was used to estimate variability within the four species except *Ursus americanus*; nucleotide divergence (i.e., average number of nucleotide substitutions per site between two sequences representing different species) [64,68] was used to assess variability between the 10 species pairs. There was no K2P correction for distances in DnaSP v5.10 [63,64]. Sliding-window analyses, which are convenient for graphical visualization, were used with a window size of 600 bp and a step size of 5 bp following Roe and Sperling [28].

**Table 2:** Mitochondrial genome sequences used in the sliding-window analyses

Species	Accession numbers
*Ursus americanus*	NC_003426
*Ursus arctos*	EU497665, NC_003427
*Ursus maritimus*	AJ428577, NC_003428
*Ursus spelaeus*	FN390857, FN390860, FN390862, FN390869, FN390845, NC_011112, EU327344
*Ursus thibetanus*	FM177759, NC_009331, NC_009971, NC_008753, NC_011118, NC_011117, EF667005

The accession numbers for the NCBI Nucleotide Database are given

### Resolution at higher taxonomic levels

In addition to the species-level resolution with which DNA barcoding is primarily concerned, we chose to investigate their efficacy at higher taxonomic levels. For the 14 NJ trees produced as above, we describe one genus that had congeneric sequences representing different species (according to their taxonomic assignment in the Nucleotide Database) as a "cohesive group", if the congeneric sequences formed a monophyletic cluster. The number of cohesive groups was summed for each NJ tree to finally give a proportion of total genera in Table 1.

We also studied eutherian relationships at the super-ordinal level, as comprehensive analyses revealed that there are four subgroups of eutherians: Laurasiatheria, Euarchontoglires, Xenarthra, and Afrotheria [39,69] (Additional file 5). Similar to the multidimensional scaling (MDS) used by Hebert et al. [7], principal-coordinates (PCOORD) analysis, which makes it easier to detect grouping of deep branches with a large data set, was employed to find low-dimensional representations of the distance matrix of objects from high-dimensional space while preserving distances as much as possible [70,71]. For the 13 gene profiles (the genome profile was not included), we first used dnadist from the PHYLIP v3.69 package [72] to calculate pairwise K2P distances, because of the suitability of the matrix format from dnadist. After transforming the distance matrix into an equivalent cross-product matrix, we used a program for PCOORD (written in Python, provided by Dr. DG. Higgins's laboratory) to plot sequence objects in the three most significant dimensions, while preserving their pairwise distances. Associated eigenvalues were plotted as well. Thus, patterns of grouping could be determined by visual inspection. Similar analyses were done for amino acids of the 13 gene profiles; Kimura's distance between two sequences of amino acids was computed using the protdist program from the PHYLIP package.

### Best-fit evolutionary model and DNA saturation

To understand further the evolutionary characteristics of the 13 gene profiles, best-fit maximum-likelihood (ML) model of nucleotide evolution was selected for each gene profile by comparing values of the BIC in ModelTest v.3.7 [42,43]. Here, 847 sequences in each profile, sharing accession numbers among 13 profiles, were used in order to maintain consistency in the sampled individuals. DNA saturation was also analyzed to examine how saturation accumulates in relation to K2P distance [28,73]. Since the transition/transversion (Ti/Tv) ratio can be regarded as an indirect measure of saturation [28], we employed PAUP* v4.0b10 [53] to compute the Ti/Tv ratio through pairwise sequence comparison. Ratios of 358,281 sequence pairs were computed for 847 sequences in each profile. After converting the Ti/Tv ratio to %Ti, we plotted %Ti values against pairwise K2P distances to compare patterns of DNA saturation among the 13 gene profiles. Low %Ti was defined as being ≦50% (Ti/Tv ≦1) [28].

## Authors' contributions

Conceived this study: CZ, AL, AZ, and SLC. Performed the work and the statistical analyses: AL, AZ, YZ, and WS. Wrote codes to facilitate computation: AL and WX. Wrote the paper: AL, SYWH, SLC and CZ. All authors read and approved of the final manuscript.

## Supplementary Material

Additional file 1 **Figure of K2P distances versus intra- and interspecific comparisons from 12 profiles**. The x-axis represents K2P distance values (%) and the y-axis represents the number of comparisons. The number of comparisons indicates either the number of species compared (intraspecific comparisons, blue) or the number of genera compared (interspecific comparisons, red). Click here for file

Additional file 2 **Figure of nucleotide divergence of 10 species pairs of the genus *Ursus***. Interspecific distances of 10 species pairs (1, *U. americanus*; 2, *U. arctos*; 3, *U. maritumus*; 4, *U. spelaeus*; 5, *U. thibetanus*) from the sliding-window analyses are shown. The x-axis represents nucleotide midpoints of the 600 bp window. The range of each gene is annotated with a pair of ticks. Click here for file

Additional file 3 **Figure of grouping results from PCOORD for 12 gene profiles**. From A to L, the figures show the grouping based on nucleotide sequences; the others are based on amino acid sequences. Symbols indicate: Afrotheria (red circle); Euarchontoglires (grey down-pointing triangle); Laurasiatheria (green up-pointing triangle); and Xenarthra (blue square). Click here for file

Additional file 4 **Figure of %Ti values against pairwise K2P distances for 12 gene profiles**. The x-axis represents the K2P distance values, while the y-axis represents %Ti values. Click here for file

Additional file 5 **Table of information of the genome and gene sequences**. Taxon information, accession number and other information are shown for each mitochondrial genome sequence. √denotes the presence of the gene sequence in the aligned gene profile, while * denotes the absence of the sequence in the aligned gene profile. Species names given in blue font indicate extinct species. Click here for file

Additional file 6 **Figure of relationship between intraspecific distances and sample sizes**. The y-axis represents average intraspecific distances less than 3% of species from the genome profile, while the x-axis represents sample sizes for these species on a log scale. Click here for file
