# Supplementary figures and images for: Potential efficacy of mitochondrial genes for animal DNA barcoding: a case study using eutherian mammals
Source: BMC Genomics. 2011 Jan 28;12:84. doi: 10.1186/1471-2164-12-84 (PMC3042414; doi:10.1186/1471-2164-12-84)

A

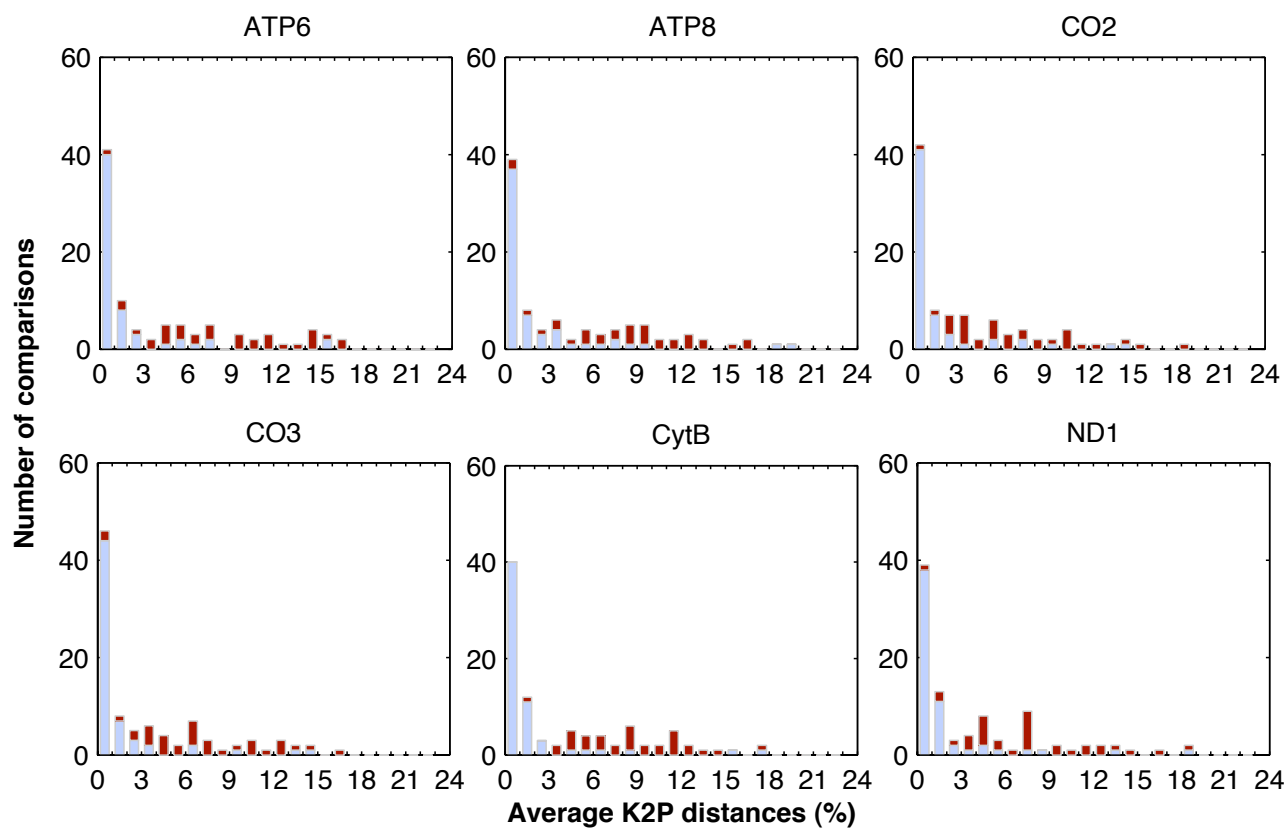

B

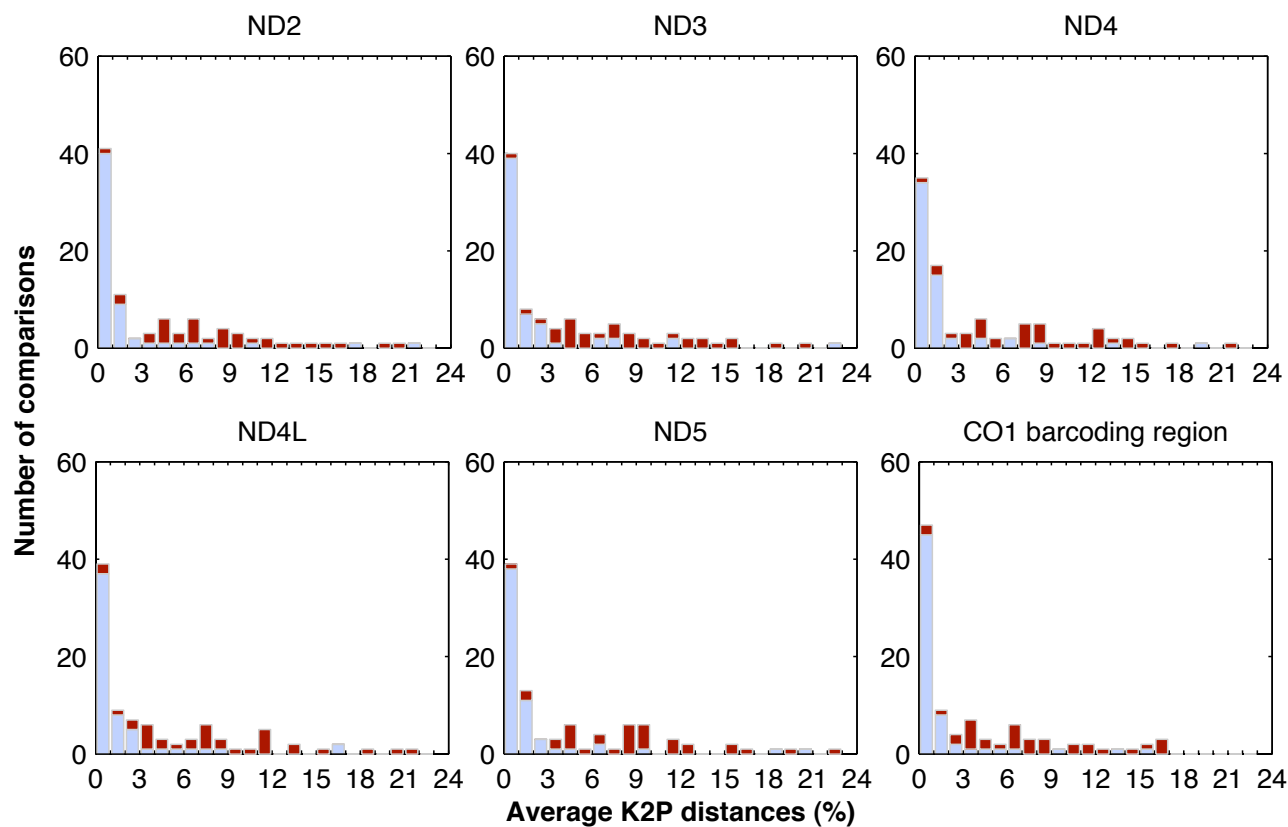

Supplement: Additional file 1 — Figure of K2P distances versus intra- and interspecific comparisons from 12 profiles. The x-axis represents K2P distance values (%) and the y-axis represents the number of comparisons. The number of comparisons indicates either the number of species compared (intraspecific comparisons, blue) or the number of genera compared (interspecific comparisons, red). [file 1471-2164-12-84-S1.PDF]

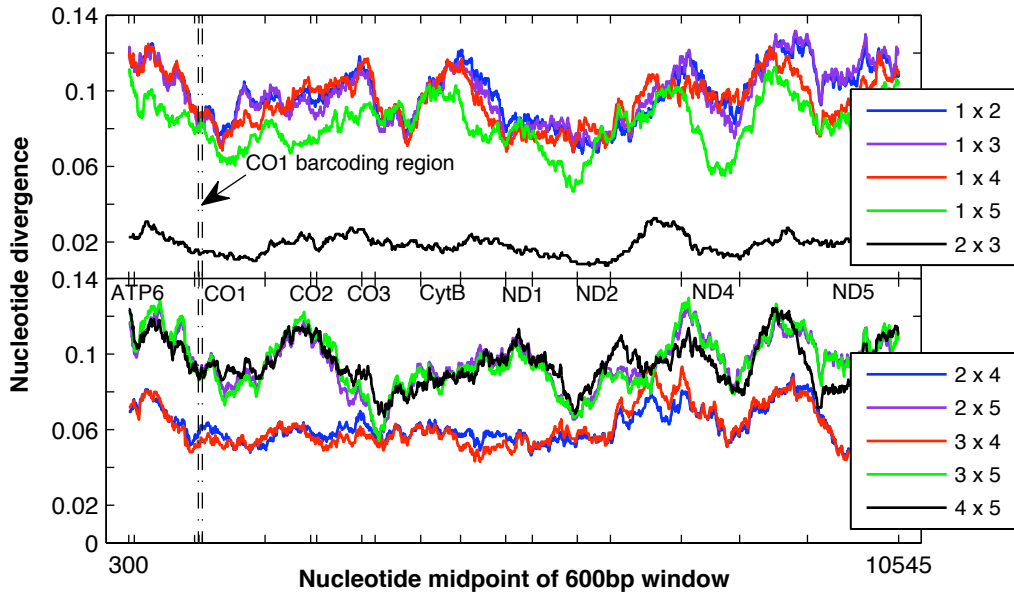

Supplement: Additional file 2 — Figure of nucleotide divergence of 10 species pairs of the genus Ursus. Interspecific distances of 10 species pairs (1, U. americanus; 2, U. arctos; 3, U. maritumus; 4, U. spelaeus; 5, U. thibetanus) from the sliding-window analyses are shown. The x-axis represents nucleotide midpoints of the 600 bp window. The range of each gene is annotated with a pair of ticks. [file 1471-2164-12-84-S2.PDF]

A

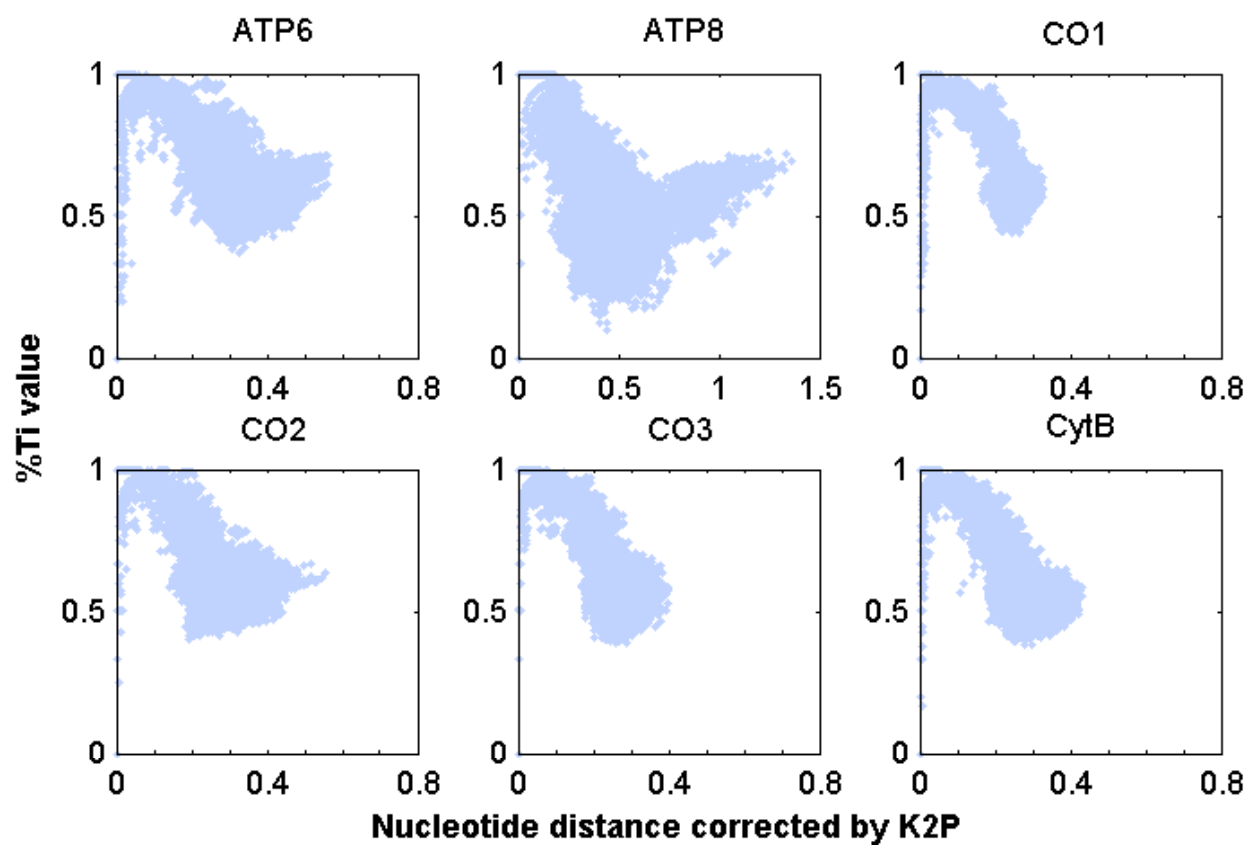

B

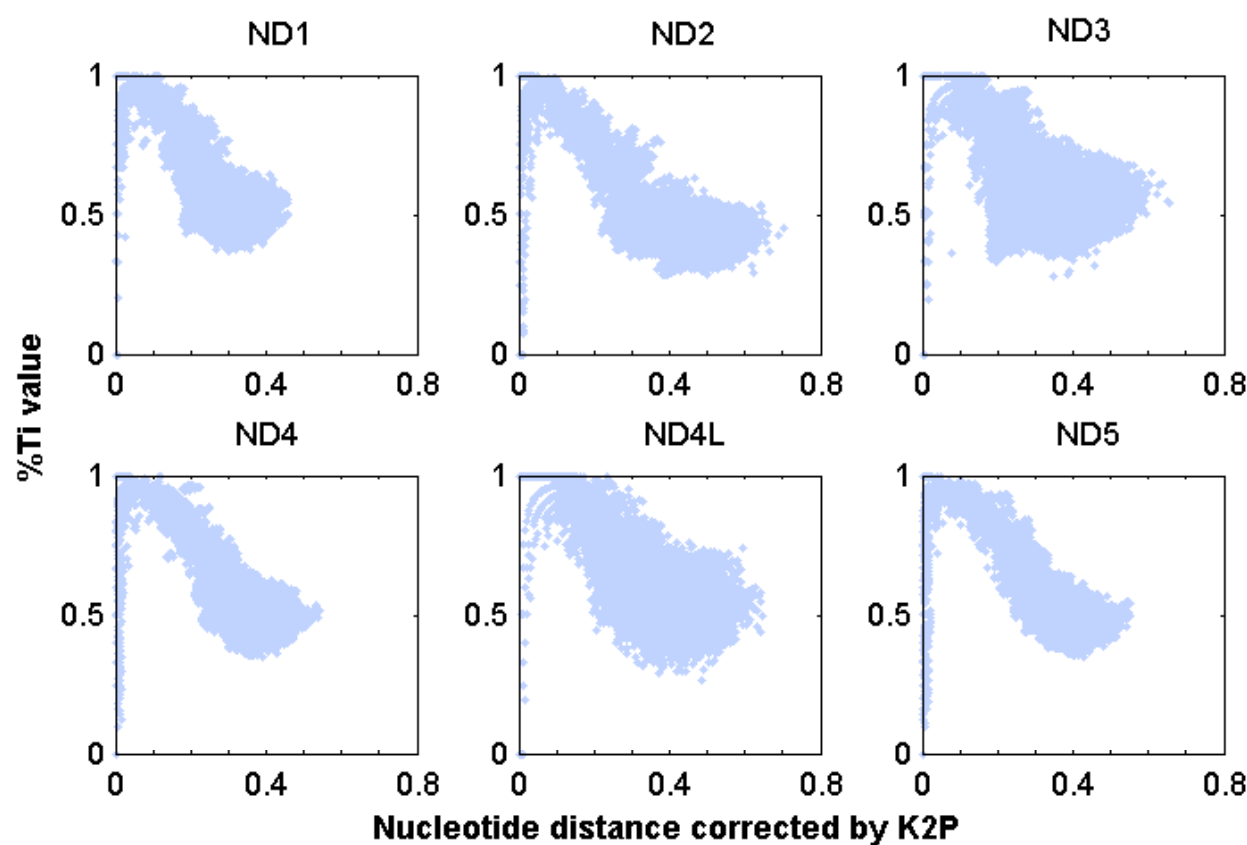

Supplement: Additional file 4 — Figure of %Ti values against pairwise K2P distances for 12 gene profiles. The x-axis represents the K2P distance values, while the y-axis represents %Ti values. [file 1471-2164-12-84-S4.PDF]

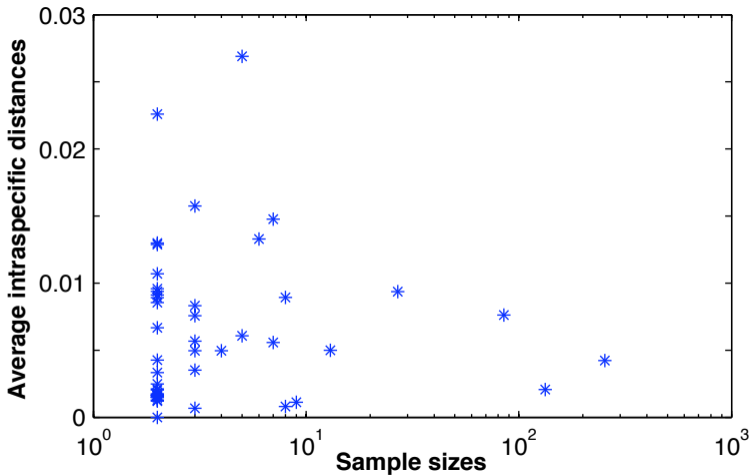

Supplement: Additional file 6 — Figure of relationship between intraspecific distances and sample sizes. The y-axis represents average intraspecific distances less than 3% of species from the genome profile, while the x-axis represents sample sizes for these species on a log scale. [file 1471-2164-12-84-S6.PDF]
