# Supplementary material for: Potential efficacy of mitochondrial genes for animal DNA barcoding: a case study using eutherian mammals
Source: BMC Genomics. 2011 Jan 28;12:84. doi: 10.1186/1471-2164-12-84 (PMC3042414; doi:10.1186/1471-2164-12-84)

A (*ATP6*, nucleotides)

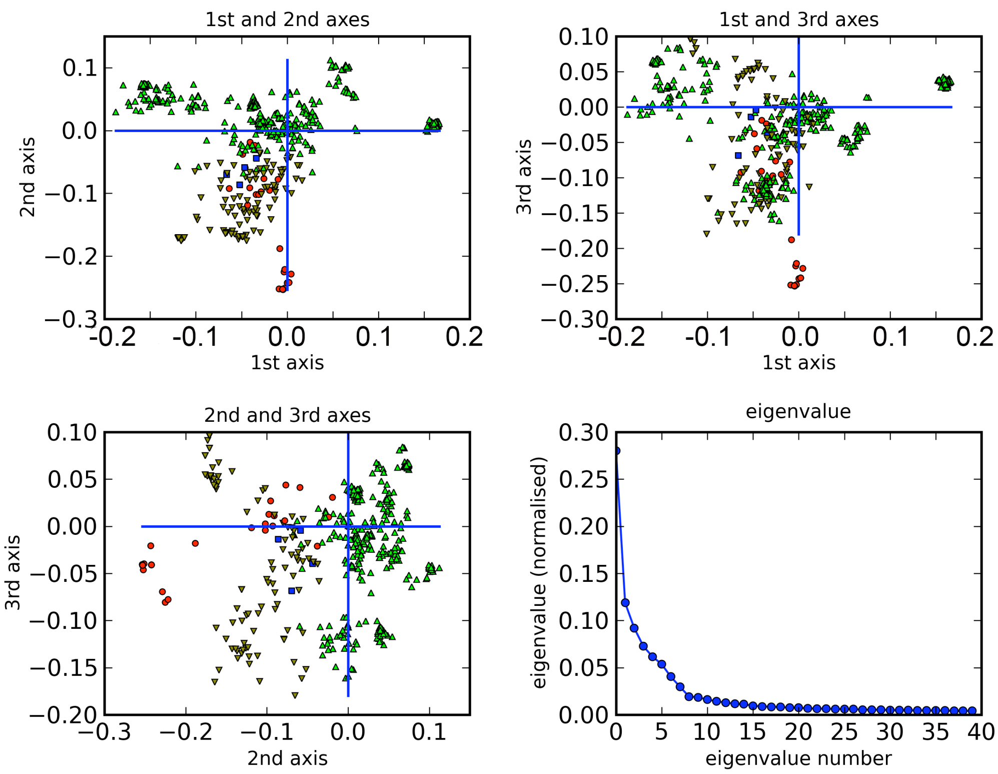

B (*ATP8*, nucleotides)

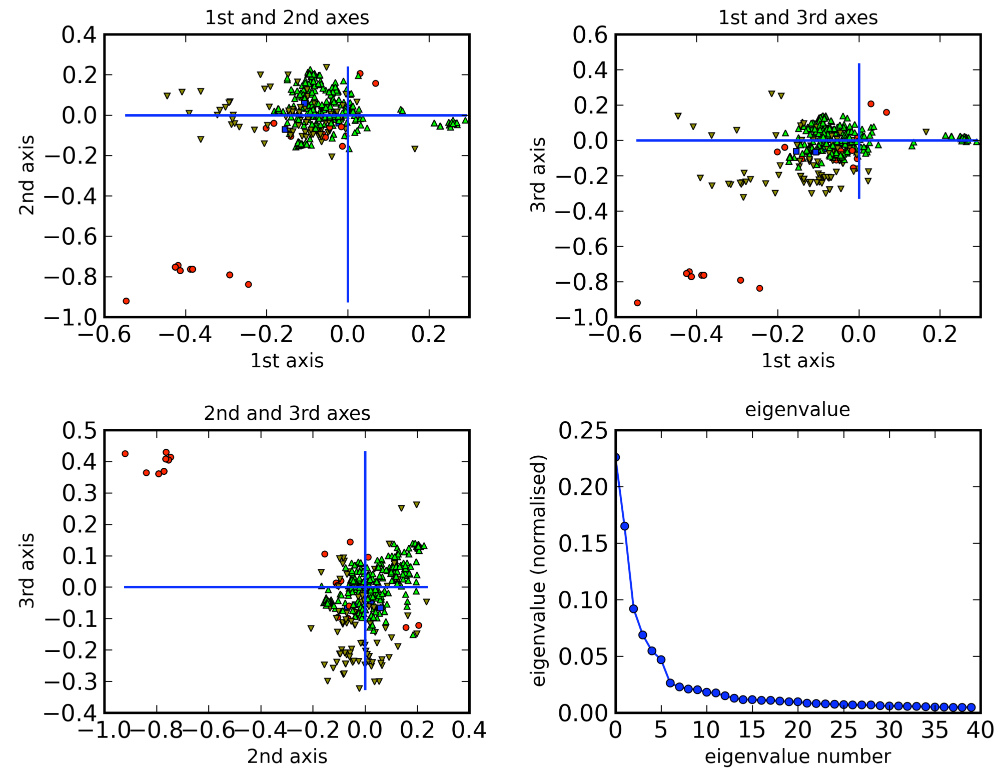

C (*CO1* barcoding region, nucleotides)

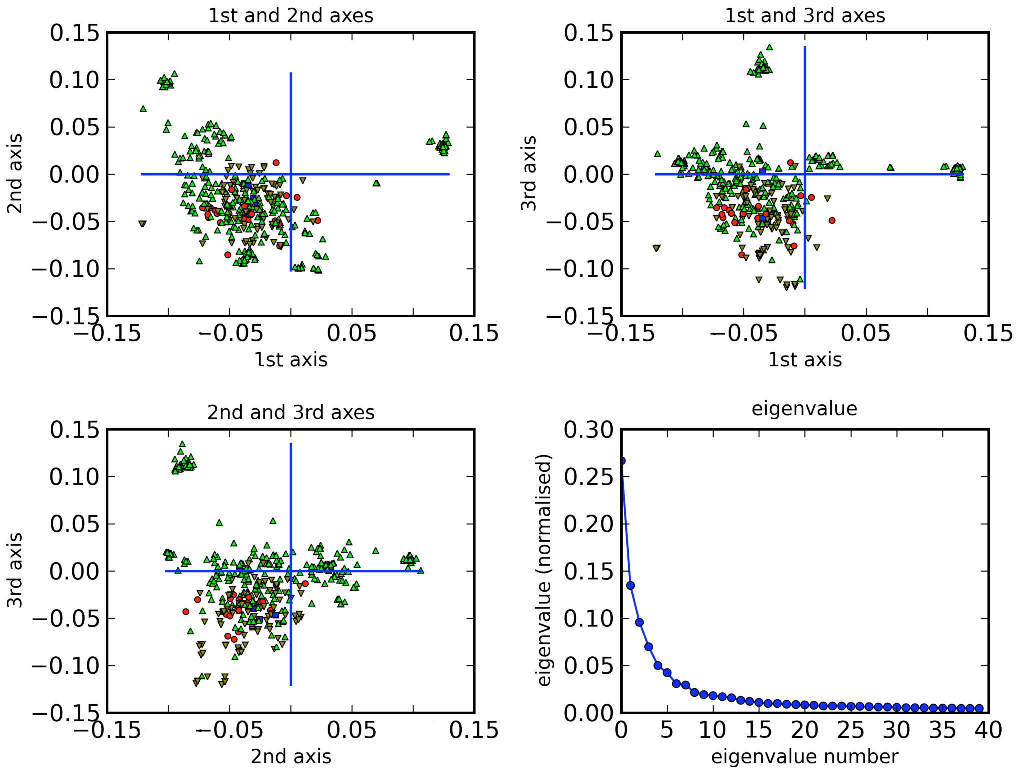

D (*CO1*, nucleotides)

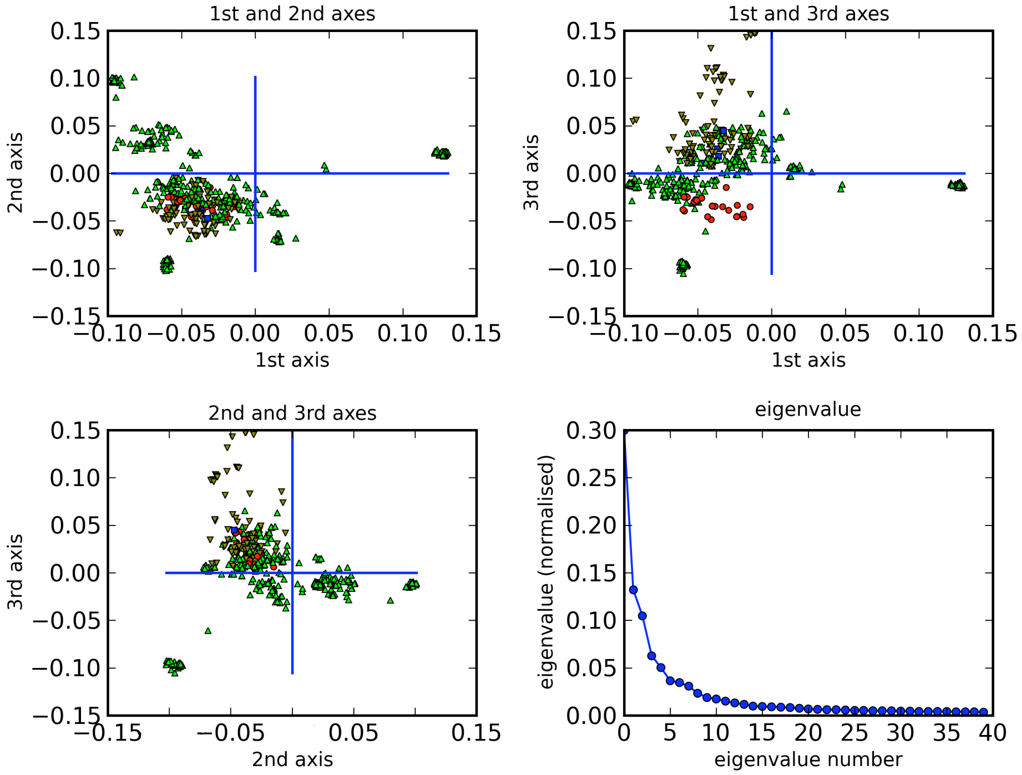

E (*CO2*, nucleotides)

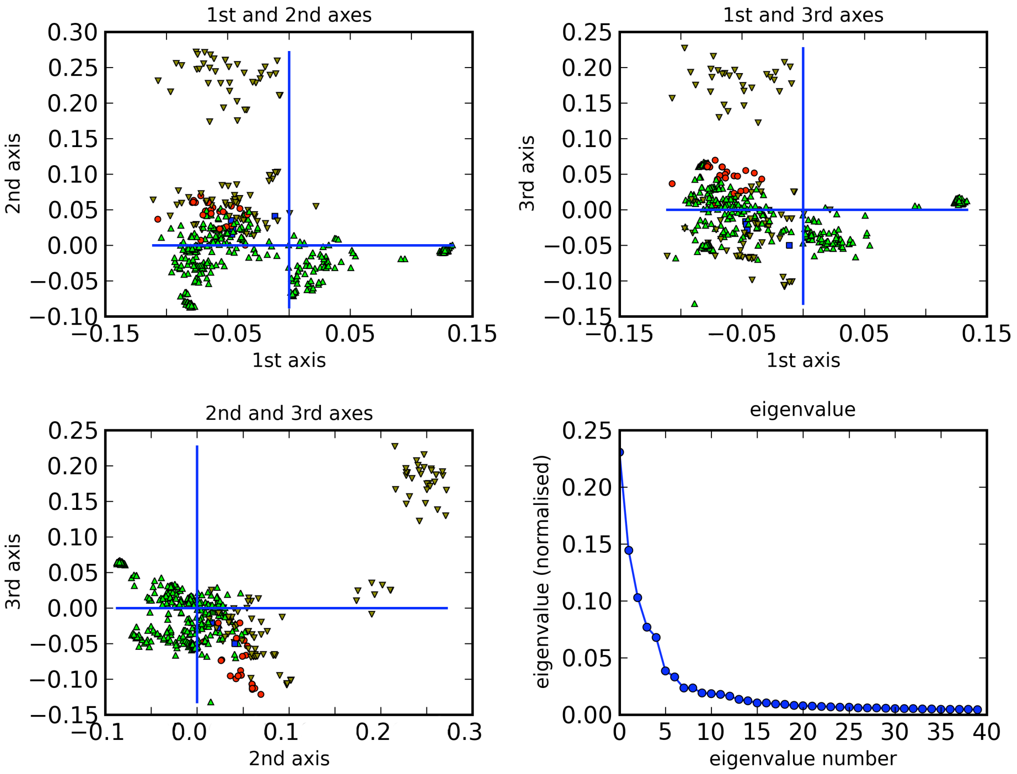

F (*CO3*, nucleotides)

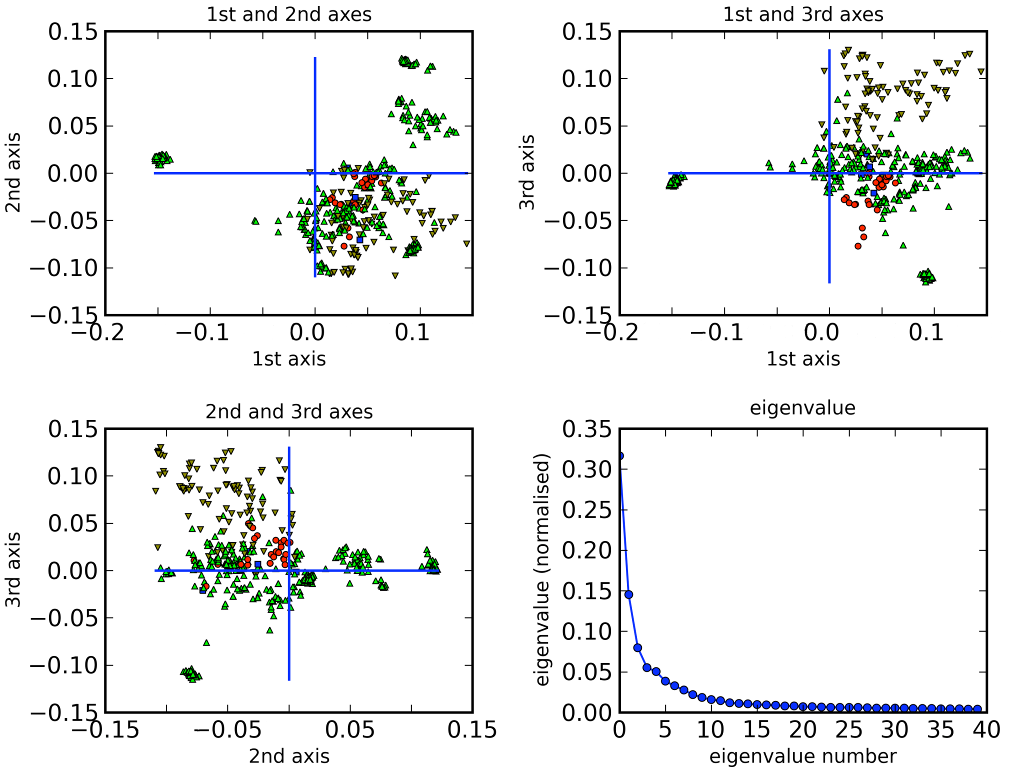

G (*CytB*, nucleotides)

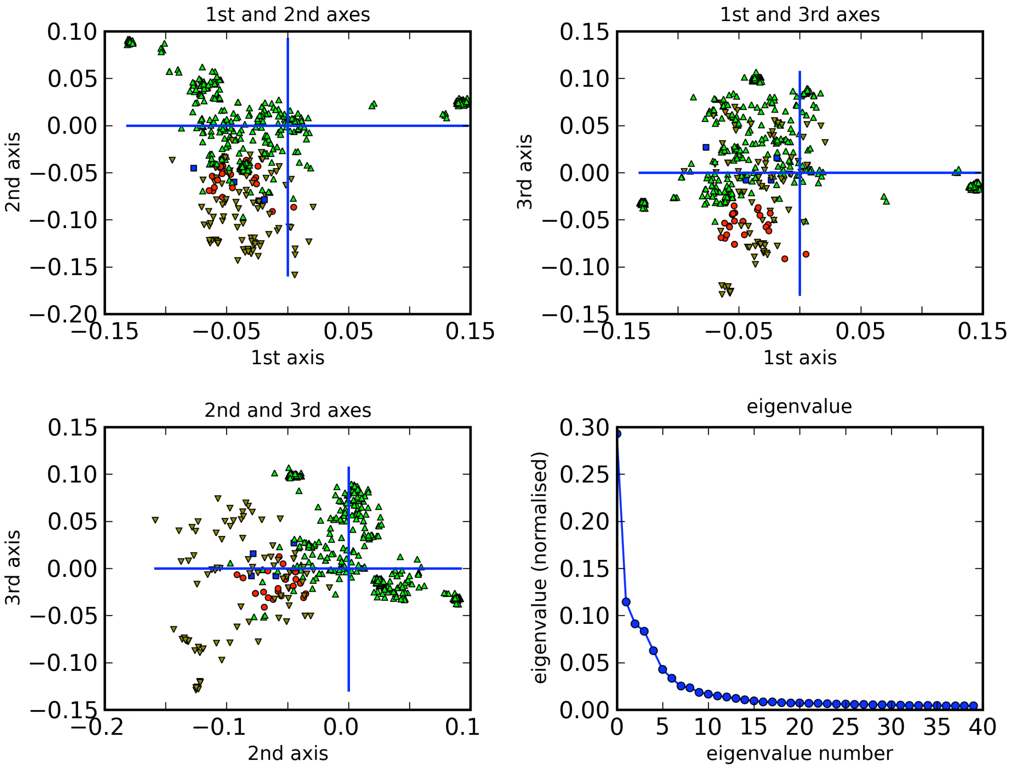

H (*ND1*, nucleotides)

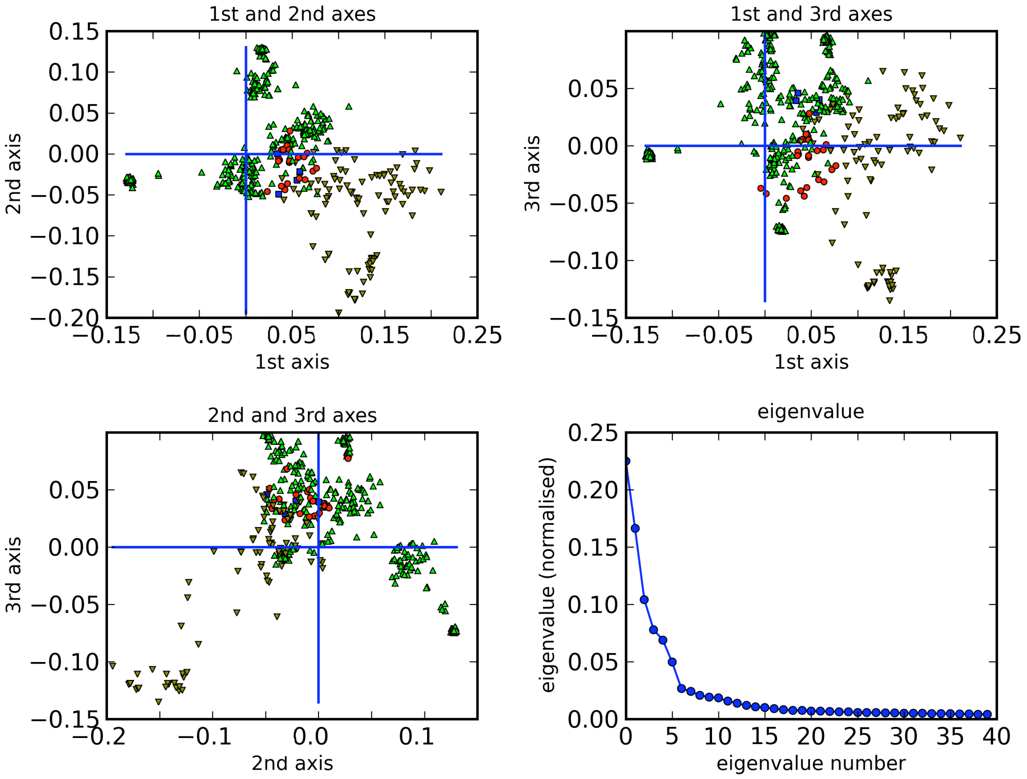

I (*ND3*, nucleotides)

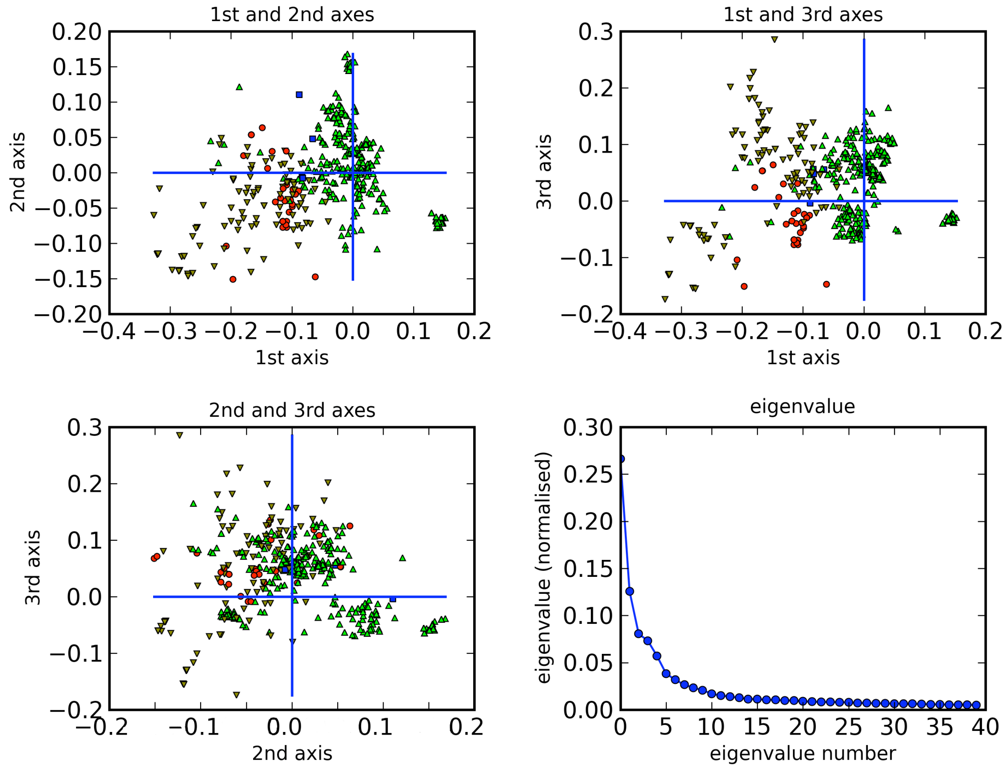

J (*ND4*, nucleotides)

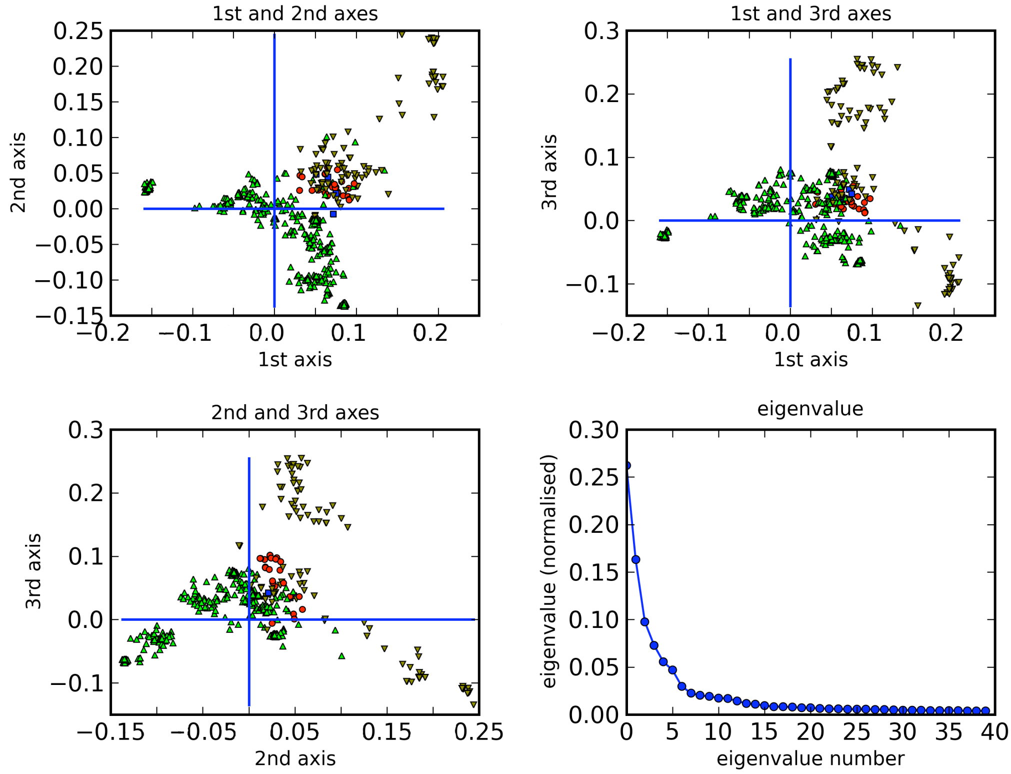

K (*ND4L*, nucleotides)

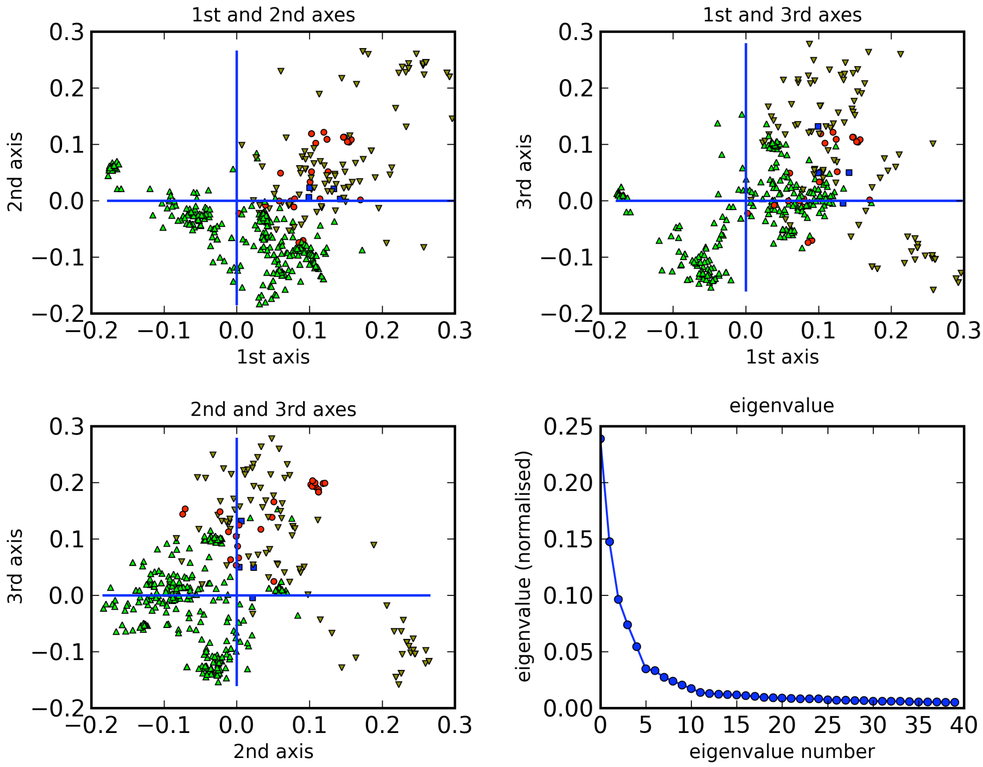

L (*ND5*, nucleotides)

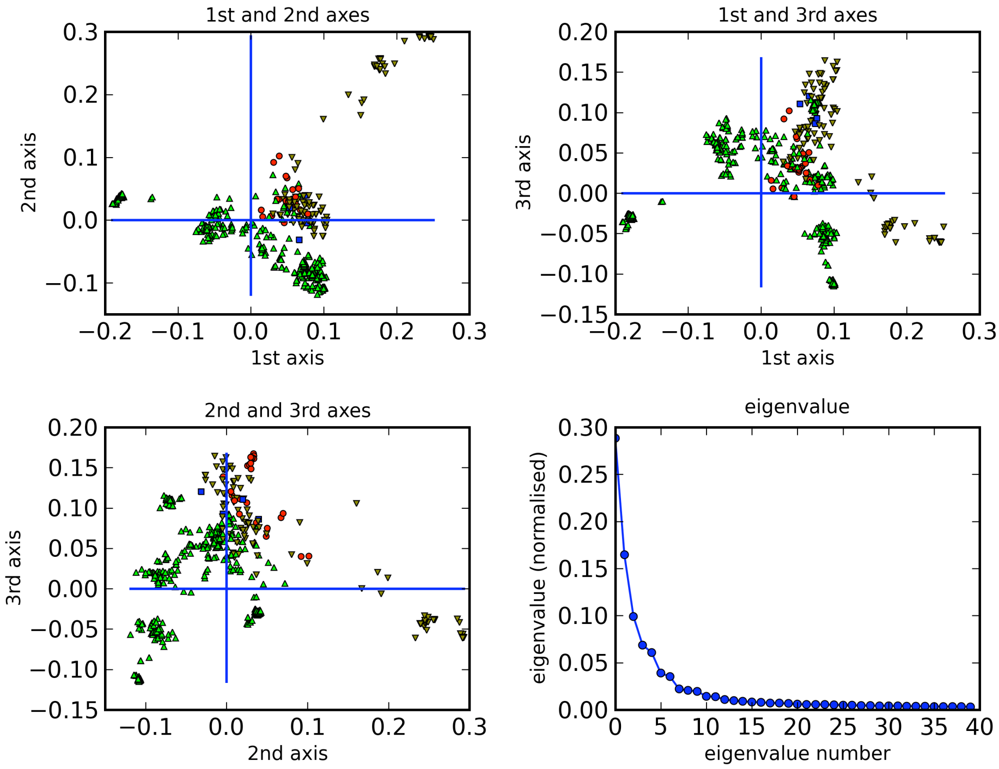

M (*ATP6*, amino acids)

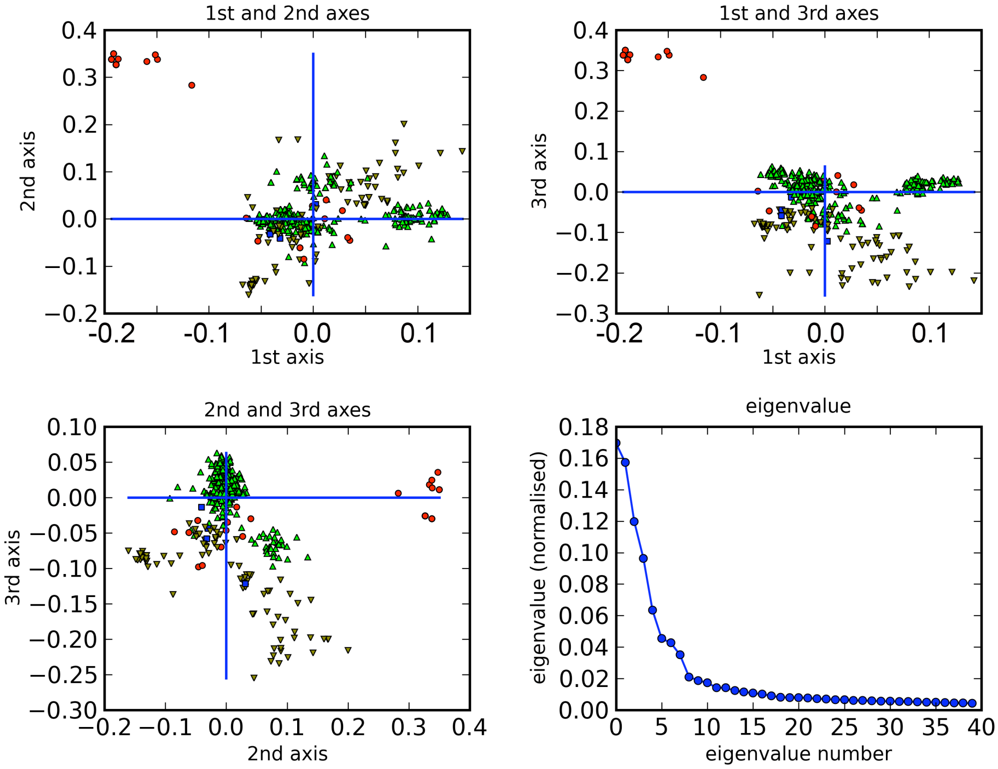

N (*ATP8*, amino acids)

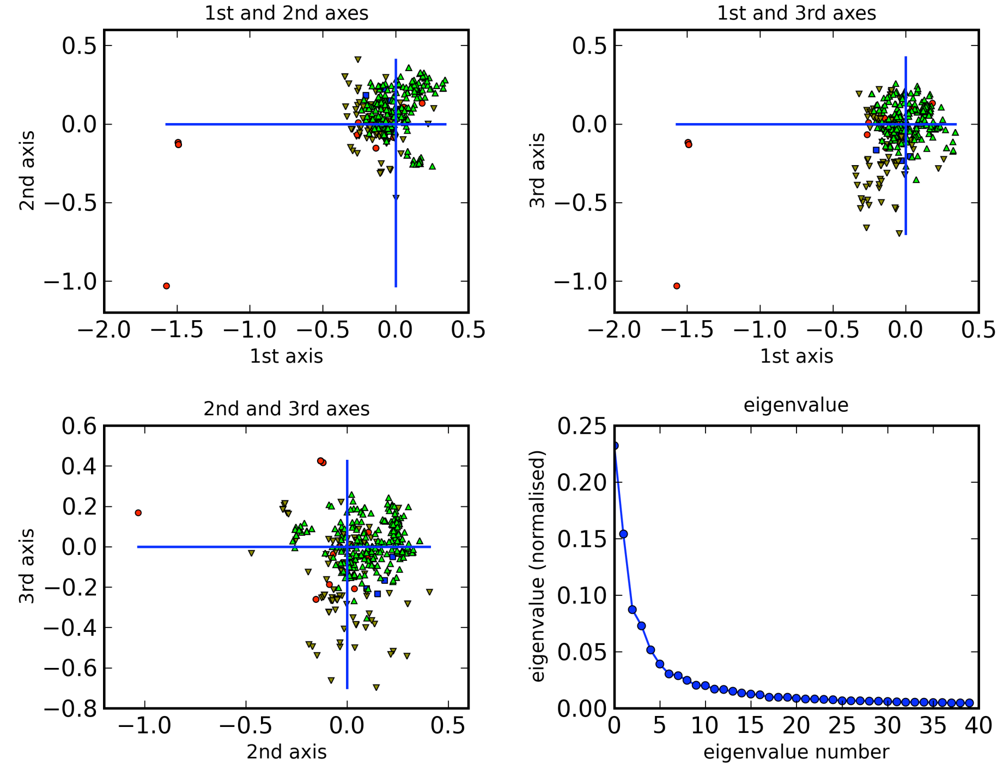

O (*CO1* barcoding region, amino acids)

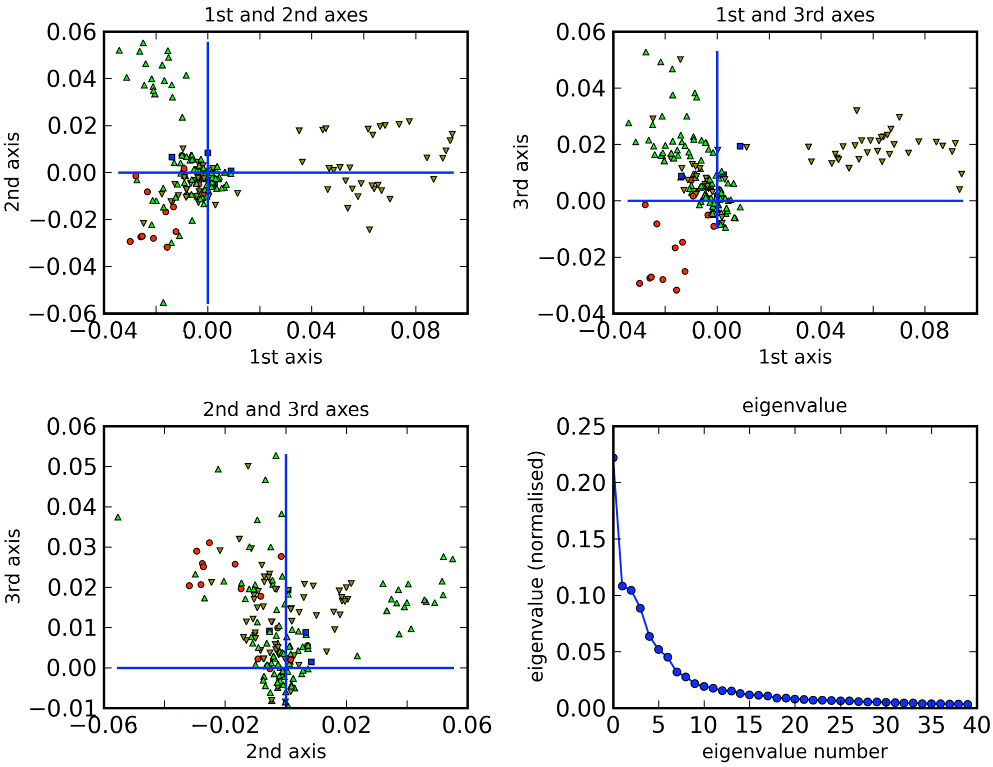

P (*CO1*, amino acids)

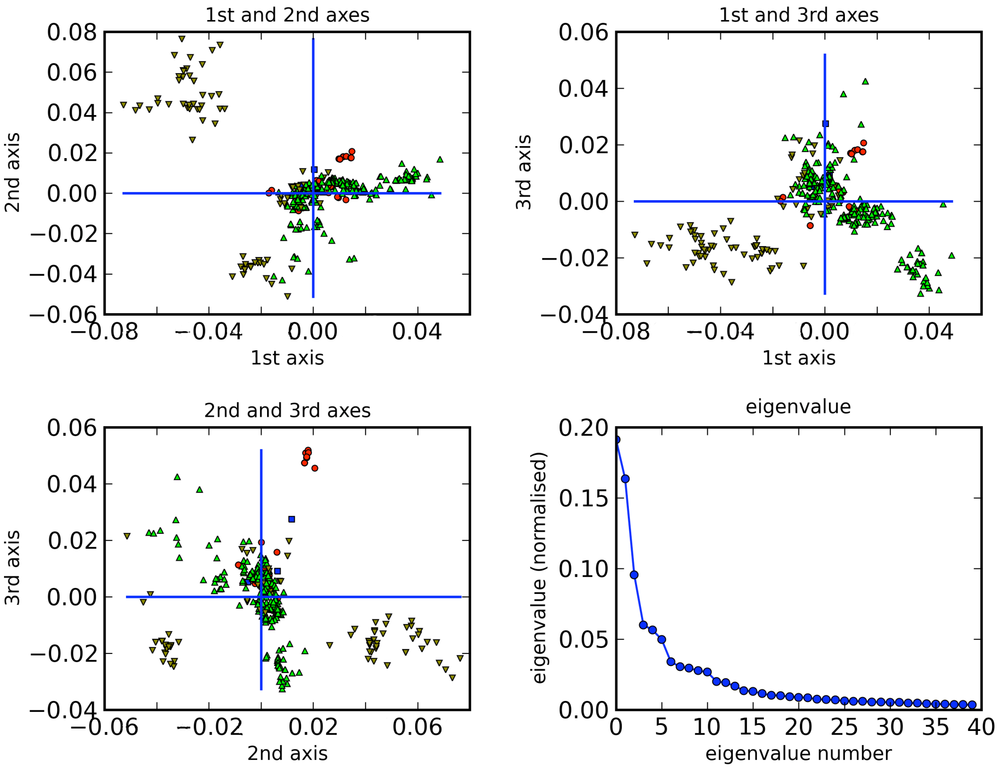

Q (*CO2*, amino acids)

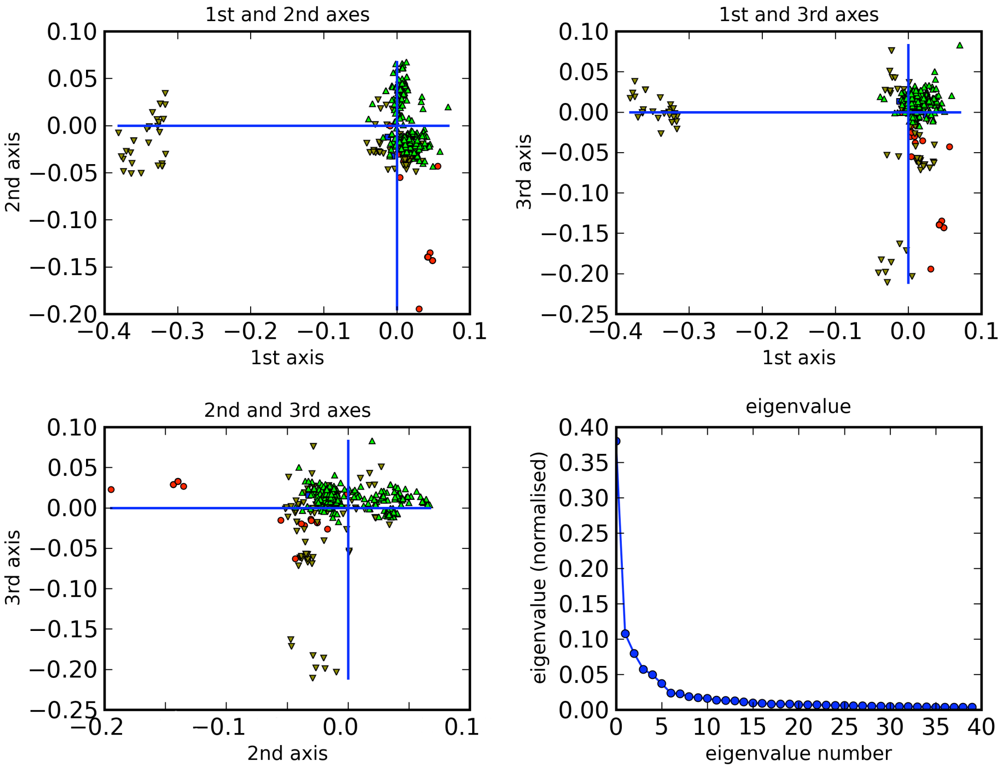

R (*CO3*, amino acids)

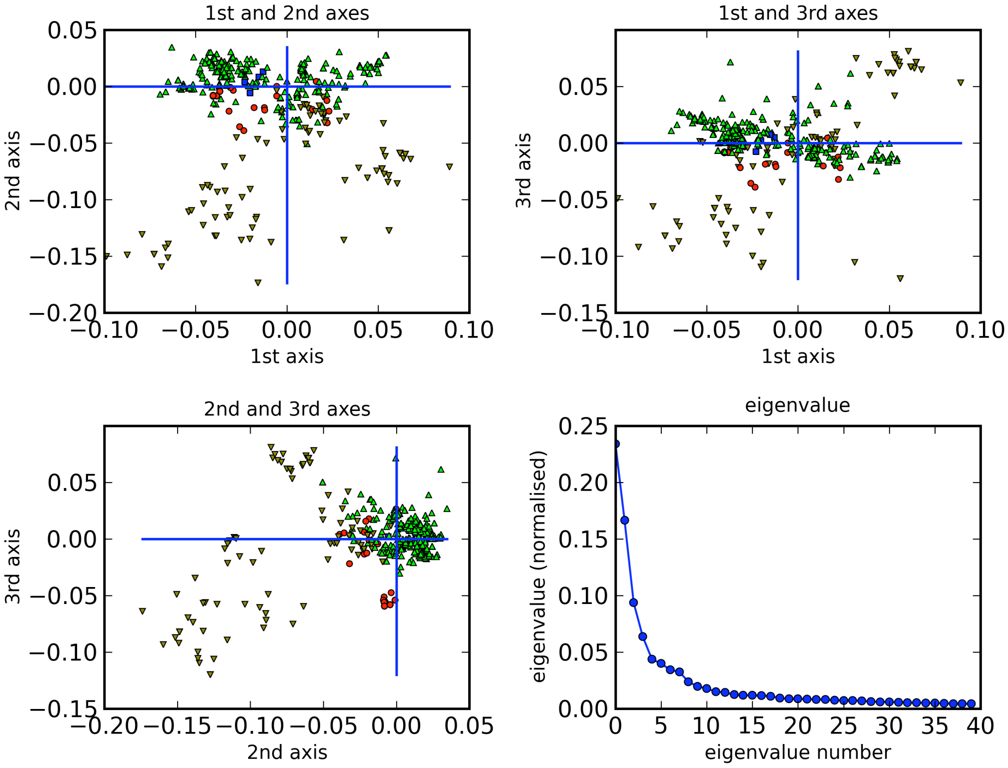

S (*CytB*, amino acids)

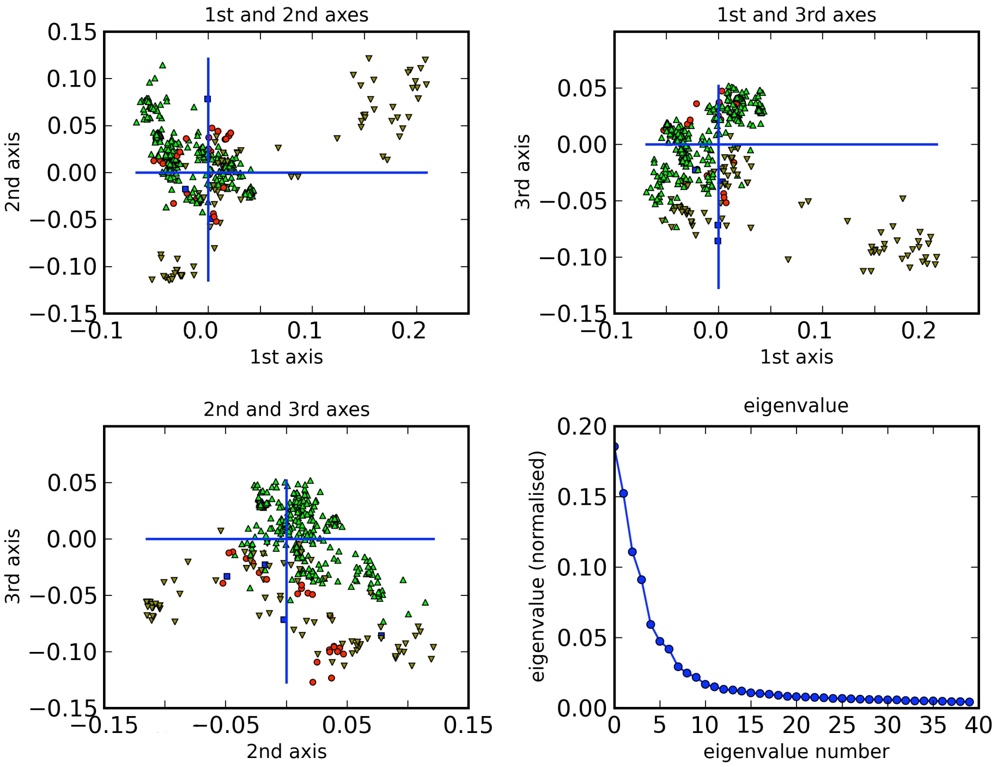

T (*ND1*, amino acids)

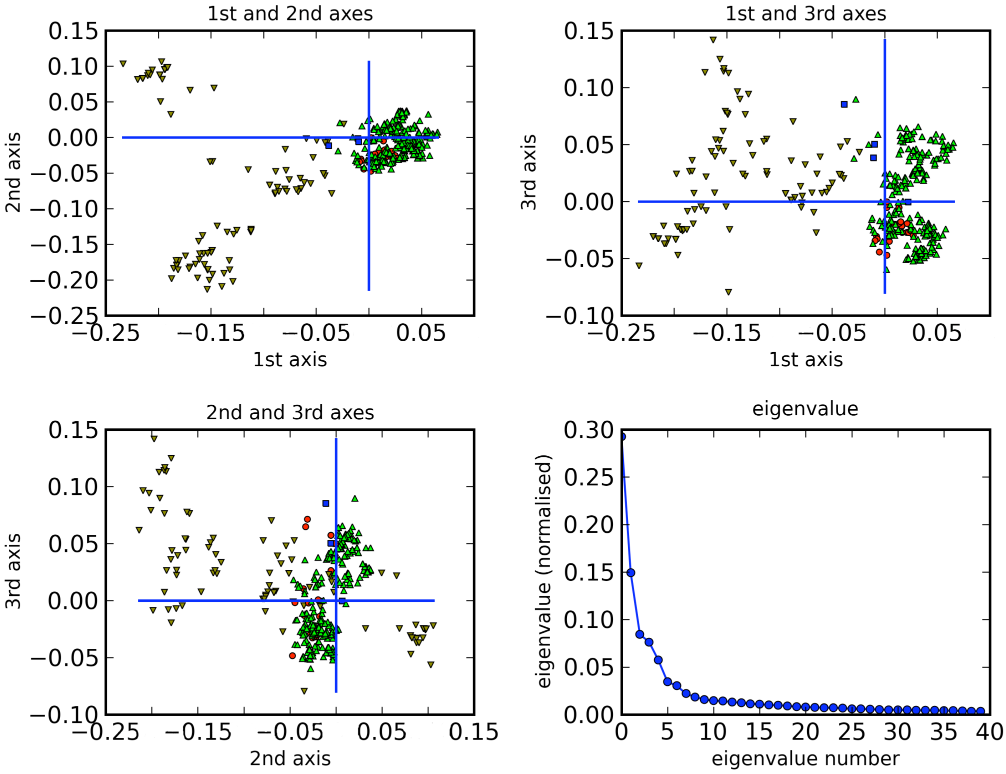

U (*ND3*, amino acids)

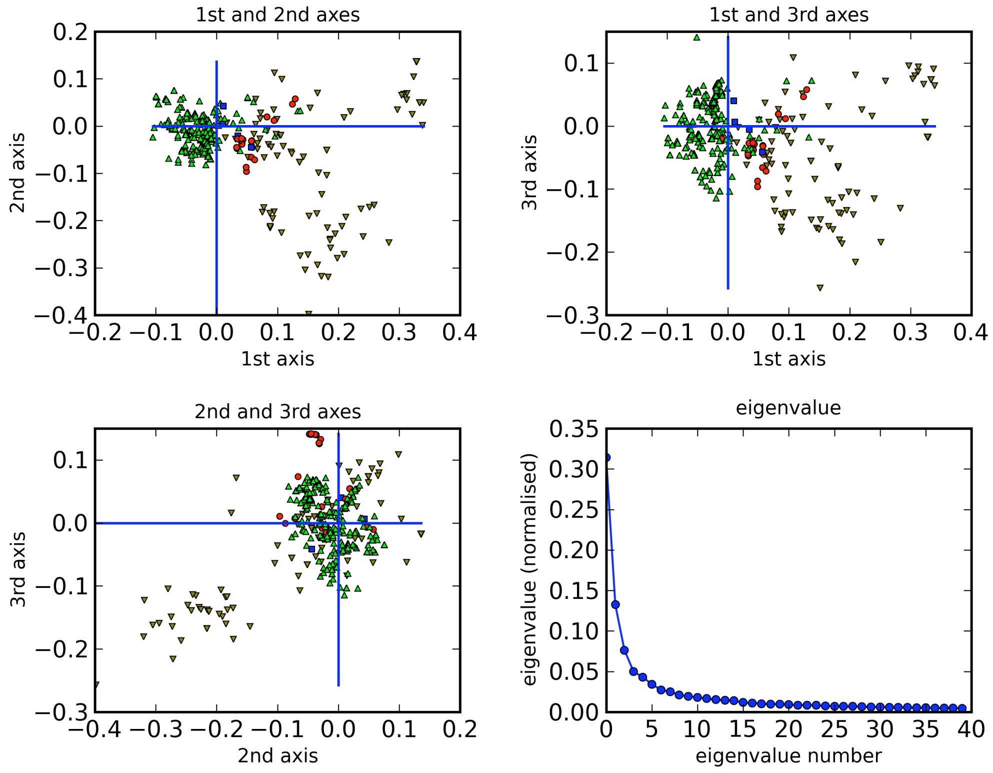

V (*ND4*, amino acids)

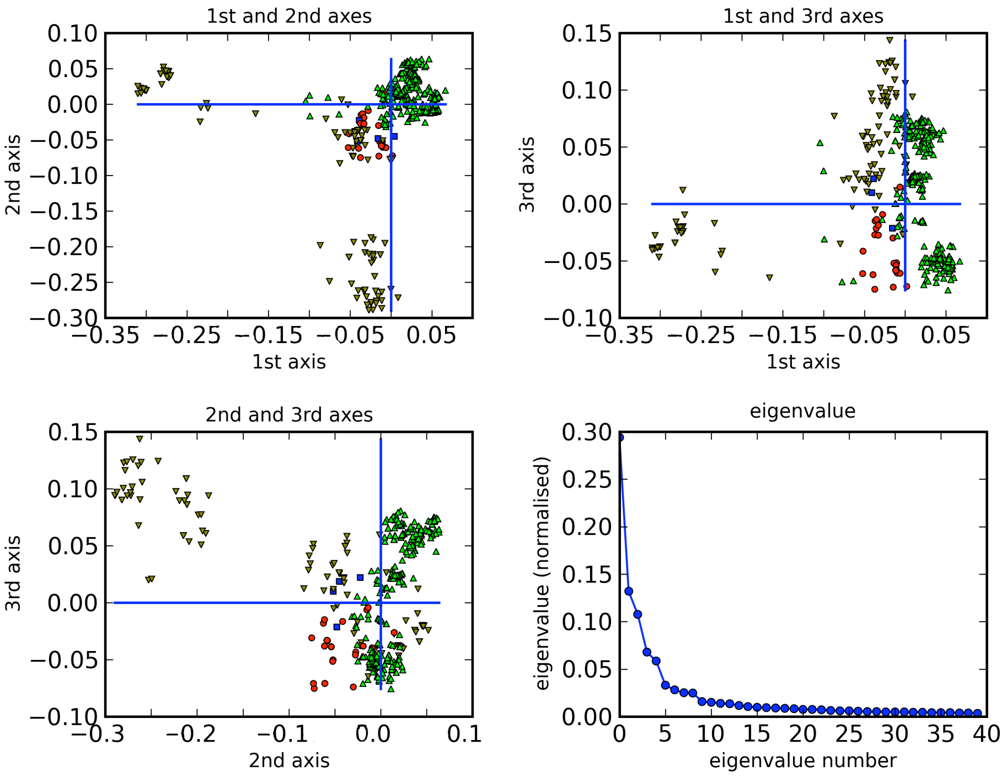

W (*ND4L*, amino acids)

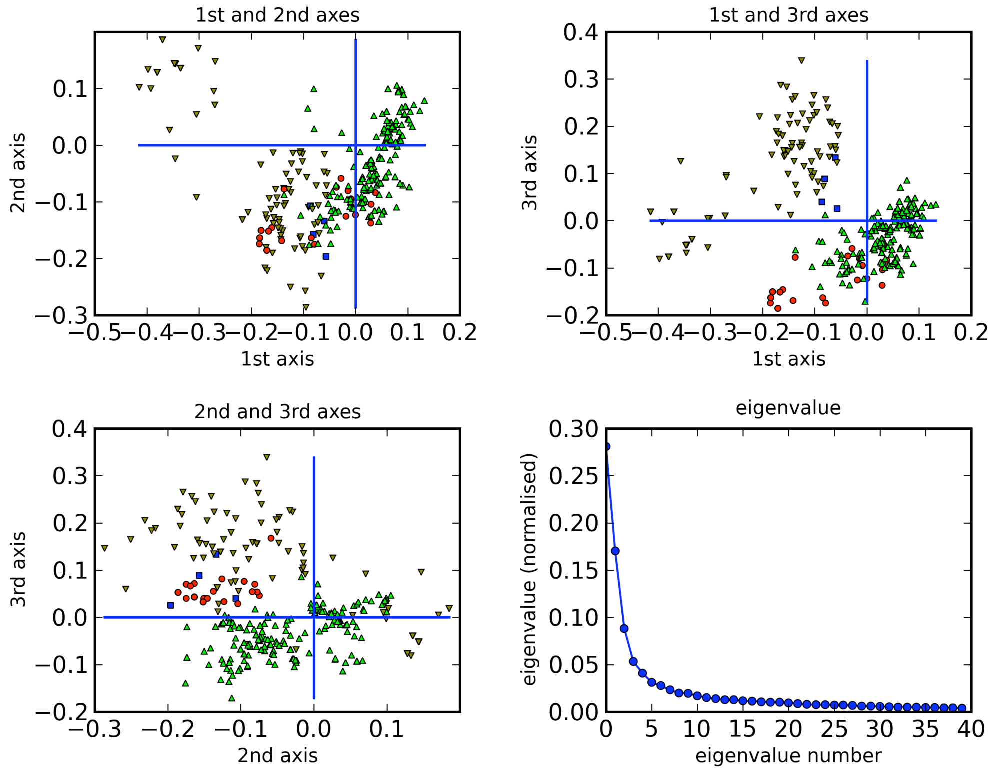

X (*ND5*, amino acids)

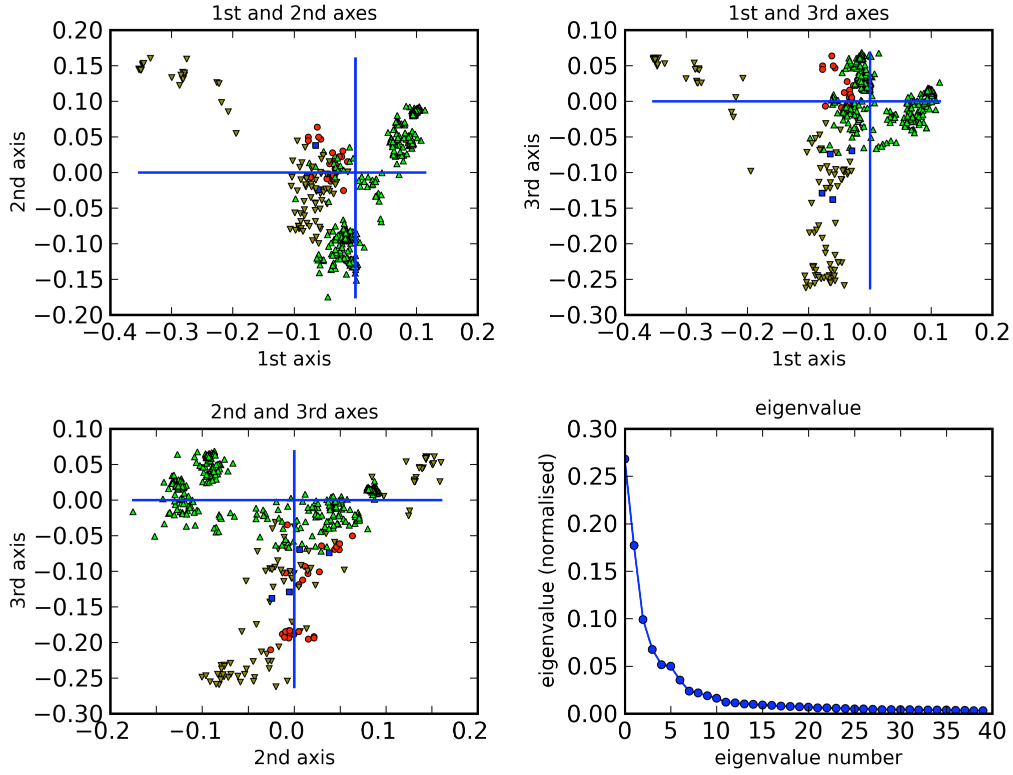

Supplement: Additional file 3 — Figure of grouping results from PCOORD for 12 gene profiles. From A to L, the figures show the grouping based on nucleotide sequences; the others are based on amino acid sequences. Symbols indicate: Afrotheria (red circle); Euarchontoglires (grey down-pointing triangle); Laurasiatheria (green up-pointing triangle); and Xenarthra (blue square). [file 1471-2164-12-84-S3.PDF]
